# Co-ordination and divergence of cell-specific transcription and translation of genes in arabidopsis root cells

**DOI:** 10.1093/aob/mcu151

**Published:** 2014-08-22

**Authors:** Dhivyaa Rajasundaram, Joachim Selbig, Staffan Persson, Sebastian Klie

**Affiliations:** 1 Institute of Biochemistry and Biology, University of Potsdam, Potsdam-Golm, 14476, Germany; 2 Max-Planck Institute of Molecular Plant Physiology, Potsdam-Golm, 14476, Germany; 3 ARC Centre of Excellence in Plant Cell Walls, School of Botany, University of Melbourne, Parkville, VIC 3010, Australia; 4 Targenomix GmbH, Potsdam-Golm, 14476, Germany

**Keywords:** *Arabidopsis thaliana*, root cells, plant cell-wall-related processes, transcriptome, translatome, system-level analysis, data integration, cell development

## Abstract

**Background and Aims:**

A key challenge in biology is to systematically investigate and integrate the different levels of information available at the global and single-cell level. Recent studies have elucidated spatiotemporal expression patterns of root cell types in *Arabidopsis thaliana*, and genome-wide quantification of polysome-associated mRNA levels, i.e. the translatome, has also been obtained for corresponding cell types. Translational control has been increasingly recognized as an important regulatory step in protein synthesis. The aim of this study was to investigate coupled transcription and translation by use of publicly available root datasets.

**Methods:**

Using cell-type-specific datasets of the root transcriptome and translatome of arabidopsis, a systematic assessment was made of the degree of co-ordination and divergence between these two levels of cellular organization. The computational analysis considered correlation and variation of expression across cell types at both system levels, and also provided insights into the degree of co-regulatory relationships that are preserved between the two processes.

**Key Results:**

The overall correlation of expression and translation levels of genes resemble an almost bimodal distribution (mean/median value of 0·08/0·12), with a second, less strongly pronounced ‘mode’ for negative Pearson's correlation coefficient values. The analysis conducted also confirms that previously identified key transcriptional activators of secondary cell wall development display highly conserved patterns of transcription and translation across the investigated cell types. Moreover, the biological processes that display conserved and divergent patterns based on the cell-type-specific expression and translation levels were identified.

**Conclusions:**

In agreement with previous studies in animal cells, a large degree of uncoupling was found between the transcriptome and translatome. However, components and processes were also identified that are under co-ordinated transcriptional and translational control in plant root cells.

## INTRODUCTION

*Arabidopsis thaliana* has become one of the most widely used plant model organisms in basic research, largely due to the availability of resources (Mochida and Shinozaki, 2010; Hamilton and Robin Buell, 2012). Recently, efforts have been made to monitor gene expression at the level of specific cell types and across different developmental stages in arabidopsis to obtain a deeper and systematic understanding of the underlying cellular processes (Edwards and Coruzzi, 1990; Birnbaum *et al*., 2005; Brandt, 2005; Shen-Orr *et al*., 2010; Wang and Jiao, 2011). These studies have resulted in the accumulation of distinct data types which provide a different, partly independent and complementary view of the whole genome. Nevertheless, to bring our understanding about plant biology to a systems level, integration of different types of data are needed (Sauer *et al*., 2007). However, integrating data from heterogeneous sources brings many challenges due to experimental, computational or statistical complexities. It is therefore also important to provide robust statistical and computational means to integrate the data (Joyce and Palsson, 2006; Zhang *et al*., 2010; Arakawa and Tomita, 2013).

The relationship between information elements (genes/transcripts) and functional elements (metabolites) has been studied by integrated transcriptomic and metabolomic analyses (Tohge *et al*., 2005; Hannah *et al*., 2010; Osorio *et al*., 2012; Brink-Jensen *et al*., 2013). In addition, there are numerous integrative studies of transcriptomic and proteomic datasets (Kleffmann *et al*., 2004; Baerenfaller *et al*., 2008; Baginsky *et al*., 2010; Vogel and Marcotte, 2012; Pan *et al*., 2012). However, these mass spectrometry-based approaches only reveal a limited coverage of the proteome and metabolome, i.e. allowing identification of a few hundred metabolites (Schauer *et al*., 2006) and a few thousand proteins in plants (Petricka *et al*., 2012). Despite the limited coverage of the proteome, recent combined transcriptomic and proteomic analyses have reported weak correlation of protein and mRNA abundances (Hack, 2004; Wang *et al*., 2010).

As an intermediate step in the flow of genetic information in a biological system, the level of translational control determines quantitative variation of the proteome together with protein degradation (Tebaldi *et al*., 2012). In particular, the composition of the translatome is based primarily on translation initiation, i.e. the loading of ribosomes on messenger ribonucleoprotein particles (mRNPs) to form polysomes, and secondarily on translation elongation (Tebaldi *et al*., 2012). Finally, correlation of levels of transcripts and polysomal-bound mRNA abundances allow inferences about gene activities and the conversion of its mRNA into a protein.

Stress-dependent coordination studies in yeast and mammalian cells integrating these two system levels have been undertaken (Halbeisen and Gerber, 2009; Tebaldi *et al*., 2012; Ventoso *et al*., 2012). While a relatively high degree of coordination of transcription and translation could be observed upon different conditions of cellular stress in yeast, divergent responses of transcriptome and translatome were found in mammalian cells. Interestingly, in yeast, harsh stresses which lead to an arrested cell growth display conserved transcriptional and translational responses, whereas relatively mild stresses also display a divergence of transcription and translation. In contrast, experiments with mammalian cells that are treated with a growth stimulus in general display predominantly divergence of responses between the two system levels.

Here, we performed a comparative study of cell-specific transcripts in plants. The relative simplicity of arabidopsis root anatomy and availability of cell-specific expression profiling data from developmental zones has made it appropriate for this study (Bevan and Walsh, 2005; Benfey *et al*., 2010). Arabidopsis also serves as a powerful model system for plant cell wall research, such as the identification of cell wall biosynthesis-related genes. Moreover, arabidopsis has also been extensively used to study the root cell wall biology and understand how cell walls are developmentally controlled in different cells (Milioni *et al*., 2002; Liepman *et al*., 2010). To complement these studies, we analysed two datasets, one transcriptome and the other of the translatome, of arabidopsis root cells. We elucidated the genome-wide correlation of cell-specific transcription and translation for the majority of genes in the arabidopsis genome. We present evidence for translational prioritization of transcripts of cell-wall-related gene families and root-related biological processes.

## MATERIALS AND METHODS

### Arabidopsis root transcriptome and translatome gene expression datasets

In this study, two microarray datasets of *A. thaliana* root samples were employed characterizing gene expression and translation levels of tissues and cell types. While the first dataset comprises a discrete global map of total mRNA levels, i.e. the transcriptome (Birnbaum *et al*., 2005; Brady *et al*., 2007), the latter measured polysome-associated mRNAs, i.e. the translatome (Mustroph *et al*., 2009), in a variety of cell types and developmental stages of the root.

The two datasets were generated using different experimental protocols and originate from two different laboratories. For the transcriptome dataset, fluorescence activated cell sorting (FACS; Iyer-Pascuzzi and Benfey, 2010) of arabidopsis radial sections of root samples under the control of cell-type-specific promoters was used to profile transcript levels. Affymetrix ATH1 microarrays were used as the platform for gene expression profiling. Genome-wide transcriptomic profiles of eight green fluorescent protein (GFP)-marked cell populations with 2–3 replicates each were obtained, that in combination with a previous study of 11 microarray expression experiments yielded a transcriptomic expression atlas. The expression atlas profiles the expression of 14 non-overlapping arabidopsis root cell types targeted by 19 promoters (Birnbaum *et al*., 2003; Brady *et al*., 2007). For the transcriptome data available from Birnbaum *et al*. (2003), cell-type and tissue-specific expression was obtained by protoplasting of plant roots expressing GFP in specific cell types. The raw data [accession number GSE ID 8934 available via Gene Expression Omnibus (GEO; Barrett and Edgar, 2006)] from the radial root sections were downloaded in the form of CEL files for further analysis.

The translatome dataset was obtained by the immunopurification of ribosome-associated/loaded transcripts from arabidopsis root cells and the immunopurified mRNAs were hybridized to the Affymetrix ATH1 microarray platform. In this study, the immunopurification was extended by using developmentally regulated promoters to drive the expression of FLAG-tagged RPL18 lines allowing the generation of 21 cell-specific populations in root and shoot (Mustroph *et al*., 2009). While the complete translatome data additionally include a stress condition (hypoxia), for the following computational analysis, raw data with an accession number GSE ID 14502 comprising only root control samples were used. An overview of all promoters used in the transcriptome and translatome data together with their intended tissue specificity can be found in Supplementary Data Table S1.

Furthermore, to avoid artefacts arising from different normalization techniques, the raw data from these two datasets were pre-processed using the same normalization strategy. Here, the robust multichip average (RMA) method was used to conduct pre-processing, e.g. removal of background noise and quality control, subsequent probe summarization and adjustment by quantile normalization (Irizarry *et al*., 2003). Applying the same normalization strategy, both datasets were jointly and independently normalized to study the influence of the normalization to the final results.

Lastly, given the availability of 19 promoters in the transcriptome and 10 different promoters in the translatome dataset (see Table S1), a common set of identical cell types corresponding to identical promoters (see Supplementary Data Promoter Sequences for the nucleotide sequences of the promoters) in both datasets – namely the phloem companion cells, root vasculature, quiescent centre, cortex and non-hair cells/root atrichoblast epidermis – were identified. Moreover, because in some cases different promoters were used to drive gene expression of the same cell type on the two system levels, a mapping of promoter and target cell type was conducted using a literature survey (cf. Results).

### Analysis of similarity of cell-specific mRNA levels on the level of transcriptome and translatome in arabidopsis root cells

**Fig. 1.**
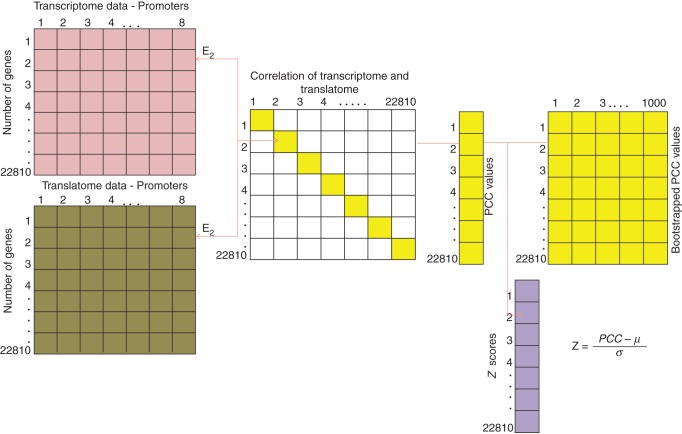
Analysis of similarity of cell-specific mRNA levels on the level of transcriptome and translatome in arabidopsis root cells using identical and common cell types for both datasets. The Pearson correlation coefficient (PCC) between expression and translation levels for each gene was computed. Eight promoters were identified that drive gene expression in common cell types in both datasets. By comparing the observed PCC value of each gene with PCC values obtained from bootstrapped data, *Z-*scores were computed for each of the corresponding genes.

Genome-scale system-level comparisons between the transcriptome and translatome were conducted by quantifying the similarity of expression (total mRNA) and translation (polysome-associated mRNA) levels of genes across the same set of cell types. Here, Pearson's correlation coefficient (PCC) was used to assess this similarity (Stigler, 1989). The statistical significance of observed PCC values was further assessed by creating 1000 bootstrapped datasets of the transcriptome and the translatome, respectively. Using the available data for all 19 and ten promoters for transcriptome and translatome as background, a bootstrapped dataset of equal size was randomly selected without replacement. As each bootstrapped group comprises a random mixture of cell types, any variations in deriving PCCs would exist mainly as a result of cell-type-specific expression rather than differences in translatome and transcriptome expression levels, thus resembling an adequate null model. For each of these bootstrapped data, and for all genes, PCC of translatome and transcriptome was calculated. *Z*-scores were calculated for each observed PCC value by subtracting the mean and dividing by the standard deviation of the corresponding gene's PCC value obtained from the bootstrapping analysis (Fig. 1). Those genes which exhibit a high positive PCC value that corresponds to a *Z*-score ≥1·96 comprise the set of genes that exhibit a high degree of correlation between transcription and translation. Likewise, PCCs of high negative value, i.e. a corresponding *Z*-score of ≤−1·96, correspond to genes that display a high degree of uncoupling of cell-specific transcription and translation. Note that an absolute *Z*-score of 1·96 corresponds to a statistical significance level of 5 % in the case of a two-tailed test (Sokal and Rohlf, 1995). Additionally, following suggestions of Huttenhower *et al*. (2006), PCC values were adjusted using Fisher transformation, resulting in normal distributions of PCC values irrespective of the dataset analysed, further allowing for cross-dataset comparisons.

Finally, a characterization of the biological processes of these genes was conducted by gene set enrichment analysis (GSEA; Subramanian *et al*., 2005). Here, the gene ontology (GO) was used to obtain functional gene annotation used for GSEA (Ashburner *et al*., 2000). Specifically, the sub-ontology *Biological Process* (GO-BP) was used to derive overrepresented GO terms and further pre-processed following the considerations given by Klie and Nikoloski (2012). For statistical testing, the hypergeometric distribution was used to test for the probability that a specific set of genes is annotated with the same GO term by considering the background distribution of GO terms (Rivals *et al*., 2007).

### Identification of altered regulation of gene expression on both system levels

In addition to the analysis of cell-type-specific gene expression/translation similarity by means of PCC, the genome-wide co-expression structure for each gene was investigated by deriving co-expression networks (Butte and Kohane, 2003). Specifically, both a co-expression and a co-translation network were constructed based on the common cell-type-specific transcriptome and translatome data to further identify the co-expression/co-translation relationships of genes which differ between the two networks. In the co-expression and co-translation networks, nodes correspond to genes and edges (connections) are present between any two nodes that are significantly connected resulting in a fully connected network (Chartrand, 1985). As a consequence, the similarity of a gene's neighbourhood within the co-expression and co-translation in the network reflects the extent to which expression and translation relationships of groups of genes are coupled. Accordingly, changed network topology suggests altered regulation or regulatory uncoupling of co-expression and co-translation relationships.

**Fig. 2.**
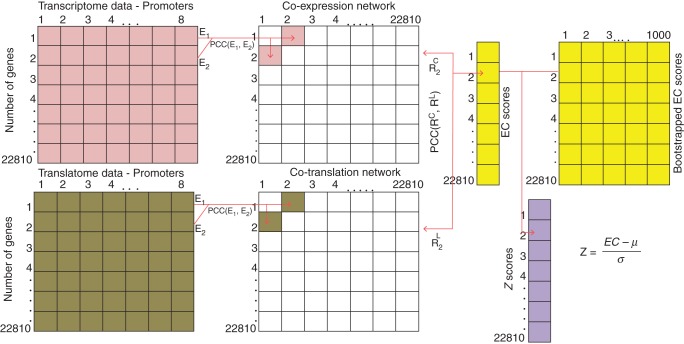
Co-expression/co-translation networks for five cell types using the common eight promoters. Two fully connected co-expression/co-translation networks were generated separately using the transcriptome and translatome datasets. An expression conservation score (EC score) was then defined as the PCC between the co-expression and co-translation networks. Further, the same analysis was performed on a set of 1000 bootstrapped co-expression/co-translation networks generated from bootstrapped datasets. Rewired genes were identified using *Z*-scores.

All edges were weighted, where the weight of an edge adjacent to two nodes/genes corresponds to the value of the PCC between the corresponding mRNA levels of both genes. For the co-expression network this weight is defined by the PCC of expression levels; likewise, in the co-translation network, this weight is defined by the PCC of translation levels in the cell types of interest. Furthermore, the concept of expression conservation (EC) was used to assess the similarity of co-expression relationships for all genes contrasting the translatome and transcriptome networks (Dutilh *et al*., 2006). Both networks can be represented by an adjacency matrix that can then be compared. Technically, this procedure simplifies the computation of the PCC of the same row corresponding to a gene in both matrices (cf. Fig. 2).

The statistical significance of the difference of a gene's EC score was assessed by creating a series of *n* = 1000 random co-expression networks for translatome and transcriptome by selecting two random equal-sized sets of cell-type-specific translatome and transcriptome data. Based on this procedure, an empirical null distribution of random EC scores, genes that exhibit a statistically significant low EC score can be derived by calculation of *Z*-scores: genes with a positive EC score and a Z-score ≥1·96 comprise genes with highly conserved co-expression relationships. Correspondingly, genes with a low EC score (matching to *Z*-scores ≤–1·96) exhibit low or no conservation of co-expression/co-translation. Again, to characterize processes overrepresented within those genes with low and high EC scores, a subsequent characterization of biological processes, GO-BP by GSEA, was conducted.

To investigate co-expression and co-translation relationships on a global scale, we computed the correlation of the adjacency matrices of both networks. The similarity of the co-expression and co-translation network is defined by their topology: as the set of nodes, i.e. genes, is the same, we compared the distribution of edges and edge-weights. As with PCC, the value of this full matrix (or network) correlation falls in the interval [–1,1] (Swanson-Wagner *et al*., 2012). Again, the statistical significance of the observed value of the full matrix correlation was assessed by selecting random cell-type expression and translation data to generate 1000 pairs of networks. Subsequently, the observed similarities were then compared with values obtained from these bootstrapped networks.

### Analysis of cell type specificity of gene expression in transcriptome and translatome of arabidopsis root cells

Differentially expressed/translated genes (for simplicity jointly referred to as DE genes) displaying statistically significant mean differences in expression levels across the common cell types were identified by performing an analysis of variance (ANOVA; Kerr *et al*., 2000). The ANOVA was performed independently for the transcriptome and translatome data. Moreover, while an ANOVA identifies genes exhibiting significant mean expression differences across all cell types, post-hoc tests, such as Tukey's honest significant difference (HSD) were applied to further derive statistical significance of pairwise mean differences (Tukey and Braun, 1994). In this study, a series of Tukey's HSD tests were performed for those genes determined to be differentially expressed by ANOVA after a Benjamini and Hochberg false discovery rate (FDR) correction (Benjamini and Hochberg, 1995). The significance level was set to 5 % for both ANOVA and Tukey's HSD.

To summarize the pairwise differences of cell type expression levels on translatome and transcriptome, cell type similarity networks were constructed for each gene. In this cell type similarity network, nodes correspond to the respective cell type of the arabidopsis root. An edge between two nodes indicates no significant mean difference of expression values of the investigated gene. Accordingly, the absence of an edge indicates a significant difference and thus dissimilarity of the adjacent nodes. Given a certain number, *n*, of cell types, a total of *m* = *n* × (*n* – 1)/2 edges can be placed between nodes resulting in 2*^m^* possible network topologies or configurations, which are defined as network motifs (Milo *et al*., 2002). Furthermore, the statistical significance of a particular number of occurrences, i.e. the number of genes that coincide with any particular network motif, was assessed empirically by permutating the data obtained for pairwise cell type mean expression differences (i.e. the results of the Tukey HSD tests) for all DE genes.

All genes identified at any step of the analysis are available in the Supplementary Data Gene List.

## RESULTS

### Promoter/cell type mapping and normalization of common transcriptome/translatome datasets

In this study, we attempted to compare and assess the relationship between cell-type-specific transcriptome and translatome data of arabidopsis roots. In particular, we were interested in testing to what degree gene expression and translation patterns were conserved in these samples. The obtained raw data were jointly pre-processed and normalized using RMA (Supplementary Data Fig. S1) to allow comparisons between the gene expression and translation datasets (Irizarry *et al*., 2003). For the transcriptome datasets, data from Birnbaum *et al*. (2005) and Brady *et al*. (2007) were used. Here, 19 cell- or tissue-type-specific root promoters driving GFP had been used in combination with cell sorting to obtain a transcriptome map of root cells. For the translatome datasets, data from Mustroph *et al*. (2009) were used. In this study, ten cell- or tissue-type-specific root promoters driving a FLAG-tagged RPL18 to achieve a translatome map of the root cells (the full list of promoters is available in Supplementary Data Table S1; see Material and Methods for further information). First, we considered the effect of separate RMA normalization of the datasets. We observed only slight differences in the distributions of probe log-intensities over all microarrays belonging either to the transcriptome or to the translatome datasets (Supplementary Data Fig. S2) indicating comparable average signal intensities between datasets and limiting the possibility of technical bias between the two datasets. We also affirmed high reproducibility of the biological replicates (correlation between replicates of 0·96 ± 0·03 in the transcriptome and 0·98 ± 0·01 in the translatome; Supplementary Data Fig. S3).

Four identical promoters had been used to obtain the transcriptome and translatome data (Birnbaum *et al*., 2003; Brady *et al*., 2007; Mustroph *et al*., 2009) and these therefore served as a first platform for our study (Table 1). Additionally, eight different promoters that target the same five cell types were also used in these two studies (Table 1). For example, *WOL* and *SHR* promoters are both indicated as vasculature-related; however, it is clear that the activity of these promoters may not exactly overlap. Nevertheless, these related promoters served as a second platform for our study. Hence, two scenarios were considered: (1) only data from the four identical promoter sets were used in comparisons (referred to as ‘identical’), and (2) combined data from the four identical promoters and the eight promoter sets that presumably target the same cell types were used in comparisons (referred to as ‘common’). Therefore, the ‘identical’ and ‘common’ datasets target four and five different cell types, respectively.

**Table 1.** List of promoters and cell types common to the transcriptome and the translatome datasets

Cell type	Transcriptome	Translatome	References
Phloem companion cells	*SUC2* (At1g22710), APL	*SUC2* (At1g22710), SULTR2	(Nawy *et al*., 2005; Lee *et al*., 2006; Brady *et al*., 2007; Mustroph *et al*., 2009)
Root vasculature	*WOL* (At2g01830)	*WOL* (At2g01830), SHR	(Brady *et al*., 2007; Mustroph *et al*., 2009)
Quiescent centre	AGL42, J0571, *SCR* (At3g54220)	*SCR* (At3g54220)	(Birnbaum *et al*., 2003; Brady *et al*., 2007; Mustroph *et al*., 2009)
Cortex	CORTEX	CO2, PEP (based on whether it is meristamatic, elongation or maturation zones)	(Lee *et al*., 2006; Brady *et al*., 2007; Mustroph *et al*., 2009)
Non-hair cells/root atrichoblast epidermis	*GL2* (At1g79840)	*GL2* (At1g79840)	(Brady *et al*., 2007; Mustroph *et al*., 2009)

Identical promoters in both datasets are underlined. The promoters used in the analysis include *SUC2* (*Sucrose transporter* 2), *APL* (*Altered phloem development*), *SULTR2* (*Sulfate transporter*), *WOL* (*Woodenleg*), *SHR* (*Shortroot*), *AGL42* (*Agamous-like 42*), *JO571* (*J0571*), *SCR* (*Scarecrow*), *CORTEX* (*Cortex*), *CO2* (*Cortex specific transcript*), *PEP* (*Endopeptidase*), and *GL2* (*Glabra2*). In addition, the genomic coordinates of the identical promoters are also specified.

### Transcription and translation of cell-wall-related genes are highly correlated

**Fig. 3.**
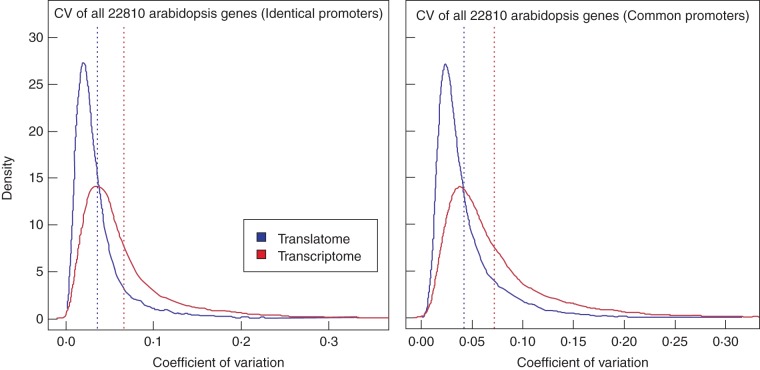
Coefficient of variation (CV) of ribosome-associated (translatome) and total mRNA (transcriptome) for the identical (left) and common dataset (right). The distribution of obtained CV values for all 22 810 genes is visualized using kernel density estimates. In the identical dataset (*n* = 22 810, bandwidth = 0·002265), the mean CV value is 0·066 and 0·036 for the transcriptome and translatome, respectively. In the common dataset (*n* = 22 810, bandwidth = 0·002568), the mean CV value is 0·043 and 0·0072 for the transcriptome and translatome, respectively. In both comparisons, the translatome displays a smaller degree of variation in cell type expression levels.

We investigated the variability in total or polysome-associated mRNA levels for any given gene to test how expression or translation patterns change across cell types. This is derived by employing the coefficient of variation (CV). Figure 3 shows the distribution of CVs for all genes (22 810 on the ATH1 platform) for the ‘identical’, and ‘common’, cell types of the translatome and transcriptome data. The transcriptome varies more (CV mean value: 0·066) than the translatome (CV mean value: 0·036) across the ‘identical’ promoter datasets. The ‘common’ promoter datasets revealed a similar scenario (CV mean value transcriptome: 0·072, translatome: 0·043).

**Fig. 4.**
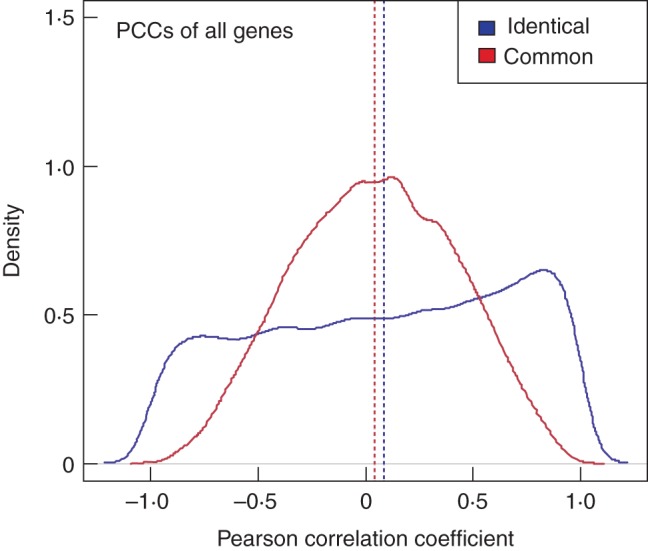
Pearson correlation coefficient (PCC) between ribosome-associated (translatome) and total mRNA (transcriptome) levels of the identical (red) and common promoter dataset (blue). The distribution of obtained PCC values for all 22 810 genes is visualized using kernel density estimates. In the identical dataset, the PCC distribution is characterized by an almost uniform shape and has a higher frequency of positive PCC values. In the common dataset, the PCC distribution resembles a normal distribution.

To examine how similar a given gene's expression and translation patterns are across the different cell types we used PCC. Figure 4 shows the PCCs between translatome and transcriptome for all genes across the ‘identical’ and ‘common’ datasets, respectively. In the case of the ‘identical’ promoter dataset, the distribution of PCCs is best characterized by an almost uniform distribution, with a slightly higher frequency of positive PCC values (mean/median: 0·08/0·12; Fig. 4). When using the ‘common’ promoter dataset the distribution of observed gene-wise PCCs resembles a normal distribution (mean = median: 0·04) in which extreme absolute values of PCCs are less common (Fig. 4).

To estimate whether the observed PCC for a gene, i.e. correlation of its expression and translation, is higher or lower then what may be observed by chance, bootstrapping was employed. Here, we re-computed PCCs using 1000 randomized datasets. Next, the observed PCC values for each gene were compared with an empirical null distribution derived from the randomized bootstrapping analysis. This null distribution of PCCs was derived by performing a bootstrap procedure randomly selecting four (for the ‘identical’ analysis corresponding to four cell types) or eight (for the ‘common’ analysis corresponding to five cell types) promoters from the transcriptome and translatome dataset (in total 19 promoters and ten promoters, respectively, see Supplementary Data Table S1). By computing Z-scores, the strength of the observed PCC value can be compared to what is randomly expected. In theory, genes with high positive or negative PCC values should therefore display high absolute *Z*-scores. Finally, based on PCC and *Z*-score, each gene can be classified into one of two groups: genes with coupled total and polysome-associated mRNA levels (high PCC and *Z*-score of ≥1·96) or genes in which the mRNA levels are *uncoupled* (low PCC and *Z*-score of ≤–1·96).

A subsequent enrichment analysis of GO-BP terms allowed us to estimate if certain processes were enriched in either of the two groups of genes. Supplementary Data Tables S2 and S3 list enriched GO-BP terms found for genes with strong positive and negative PCC values, respectively. We found that 494 and 373 genes displayed uncoupled expression and translation for the ‘identical’ and ‘common’ promoters, respectively. These genes were enriched for GO-BP terms related to cell growth, root and meristem development, protein glycosylation and cytoskeletal organization (Supplementary Data Tables S2 and S3). However, it is important to remember that the two datasets, i.e. the transcript and translation datasets, were generated in two different labs with two different techniques, and it is therefore possible that some of the uncorrelated processes are due to these differences.

We found that 851 genes and 790 genes for the ‘identical’ and ‘common’ promoters displayed coupled expression and translation, respectively. GO-BP enrichment analyses showed that these genes are associated with regulation of transcription, post-translational modification (protein phosphorylation), and responses to various biotic and abiotic stimuli/stresses (Supplementary Data Tables S2 and S3). Moreover, we found that genes associated with cell-wall-related processes and root tissue formation processes were common. This comprises GO-BP terms such as cell wall modification, secondary cell wall biogenesis, xylan biosynthetic process, xylem and phloem pattern formation, and meristem initiation. These data are in agreement with various co-expression approaches that have been undertaken for secondary wall synthesis, i.e. many secondary wall genes are transcriptionally and functionally coordinated (Persson *et al*., 2005).

### Co-expressed relationships at the transcriptional level are generally not preserved at the translational level

So far, our analysis has focused on quantifying the degree of similarity in expression and translation for individual genes across different cell types. However, one could also investigate whether larger contexts of genes are coordinated across the two levels. To assess whether genes that are transcriptionally coordinated, or co-expressed, are also coordinated on a translational level, we constructed co-expression and co-translation networks for the ‘identical’ and ‘common’ promoter datasets. Note that while a particular gene can exhibit changes between cell-specific transcription and translation, this does not exclude that the co-expression and co-translation neighbourhoods of genes are preserved, i.e. one could imagine that certain co-expressed genes change their translational patterns in a coordinated fashion.

For one particular gene, an EC score is derived by calculating the PCC between the adjacent edge-weights, i.e. the co-expression relationship, of the two networks thus capturing similarity of gene neighbourhoods (Fig. 2). Accordingly, genes displaying low EC scores show different patterns of co-expression relationships in the two respective networks, while high EC values indicate the presence of highly similar co-expression relationships on the translatome and transcriptome.  

**Fig. 5.**
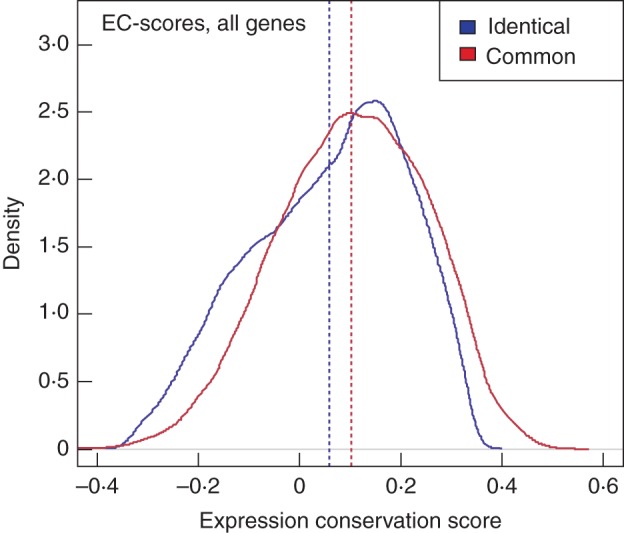
Expression conservation (EC) scores of co-expression relationships on the translatome and translatome within the identical (red) and common promoter dataset (blue). The distribution of obtained PCC values for all 22 810 genes is visualized using kernel density estimates. For both the identical and the common dataset, EC score values lie in the interval 0·4–0·5.

The edges are weighted according to the similarity of expression/translation, which is defined as the PCC scores between the cell-specific expression/translation levels of the neighbouring genes. For each gene, the EC, i.e. the similarity of the gene's genome-wide co-expression and co-translation relationships, was calculated. For this, differences in the edge-weights of a gene's incident edges, i.e. its network neighbourhood, are compared between the co-expression and co-translation networks. The computation of EC score has been previously applied to elucidate the ‘expression context’ of orthologous genes in four Eukaryote species, successfully illustrating that co-expression neighbourhoods of orthologues are highly conserved (Dutilh *et al*., 2006). Figure 5 shows the distributions of EC scores using the ‘identical’ and ‘common’ promoters, respectively. For both the ‘identical’ and the ‘common’ datasets, the range of EC scores lies in the interval–0·4 to 0·5.

**Fig. 6.**
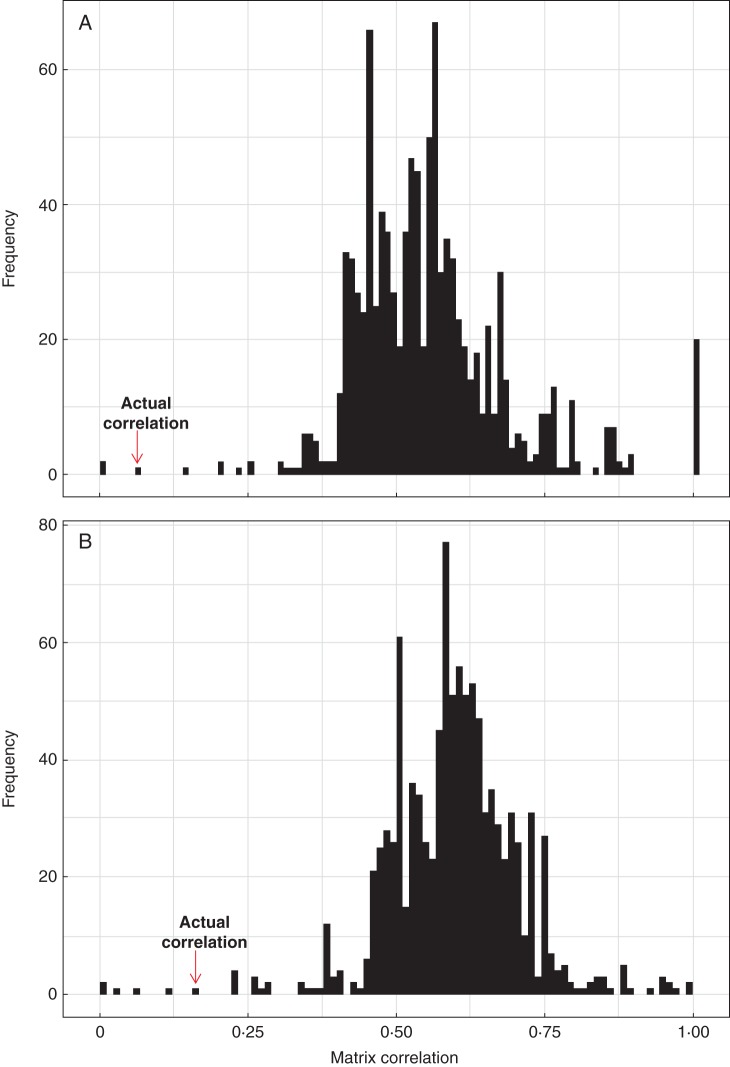
Similarity of the co-expression and co-translation network for the identical (A) and common (B) dataset. The similarity of both networks is determined by the PCC of the adjacency representation of the networks, i.e. a full matrix correlation. Only 0·001 and 0·005 % of the 1000 pairs of networks derived from bootstrapping procedure exhibit lower correlations than the observed transcriptome and translatome networks, respectively. This indicates a low degree of coupling of co-expression relationships between the two system levels.

To validate the observed relationships on the level of co-expression and co-translation networks, we derived the correlation on a global scale by considering the entire networks. The observed value of the full-matrix correlation was 0·06 and 0·10 between the co-expression and co-translation networks for the ‘identical’ and ‘common’ promoters, respectively. To assess whether these values are different from what could be expected by chance, we again selected random cell type expression and translation data, and used 1000 bootstrap samples that then were compared against our observed similarities (Fig. 6). For both the ‘identical’ and the ‘common’ promoter sets we found the values to be statistically significantly lower than expected by chance (*P* < 0·01). These data suggest that globally, or genome-wide, co-expressed gene patterns are dissimilar from co-translational patterns in arabidopsis root cells.

Returning to a per-gene analysis, we investigated which genes either significantly deviated or correlated in co-expression and co-translation relationships (i.e. EC scores) by assessing statistical significance by *Z*-scores and bootstrapping. Hence, these genes either exhibited tight coupling (high EC score, and *Z*-scores ≥1·96) or uncoupling (low EC scores, and *Z*-scores≤–1·96; Fig. 2) of their co-expression and co-translation to other groups of genes, thus reflected in conserved or changed network neighbourhoods. Only 71 and 39 genes exhibited (statistically significant) high EC scores for the ‘identical’ and ‘common’ promoters, respectively. In contrast, 10 057 and 12 681 genes displayed (statistically significant) low EC scores for the two promoter sets, respectively. Subsequently, we tested if these sets of genes were enriched for certain GO-BP terms (Supplementary Data Tables S4 and S5). For both groups of genes (high/low EC scores) and promoter sets (‘identical’/‘common’), we found enrichment of the GO-BP terms DNA-dependent regulation of transcription, cell wall biogenesis and organization, transmembrane transport, cell wall organization and cell growth, and signal transduction. Due to the high number of genes with uncoupled co-expression and co-translation relationships, i.e. low EC scores, we found numerous GO-BP term enrichments, including a wide range of metabolic and catabolic, as well as transport processes.

**Fig. 7.**
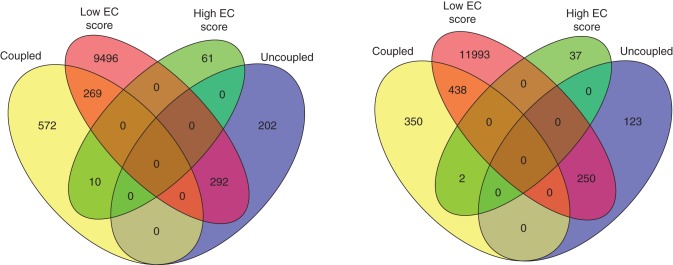
Venn diagrams illustrating the overlap of genes displaying conserved expression levels (PCC) and co-expression relationships (EC scores) across root cell types in translatome and transcriptome. Ten genes could be identified using the scenario considering identical promoters (four promoters, left) and two genes considering common promoters (eight promoters, right).

Finally, to assess what types of genes both display a good correlation between expression and translation (high PCC score above) and retain a good correlation between co-expression and co-translation network neighbourhoods (high EC score, above) we identified such genes for the ‘identical’ and ‘common’ promoter sets. Figure 7 shows that ten genes (‘identical’ promoters) and two genes (‘common’ promoters) have these characteristics (Table 2). Furthermore, a more detailed network analysis was conducted to analyse whether additional network properties of those 12 genes deviate from the majority of genes and further explain the conservation/similarity of neighborhoods. Thus, un-weighted, classical gene-relevance networks were created, using a threshold, *t* = 0·9, for the PCC of two genes to decide whether an edge (≥*t*) or no edge (<*t*) is present. Based on this threshold, two networks were created dichotomously capturing co-expression and co-translation and properties of genes across the two system levels. The network properties considered here comprise: degree, edge betweenness, closeness, Eigen vector centrality, Alpha centrality and transitivity. Interestingly, for all of the tested properties, the 12 genes display network properties that are well distributed across the whole range of the corresponding properties as compared with all genes (Supplementary Data Fig. S4).

**Table 2.** Genes displaying conserved expression levels (PCC) and co-expression relationships (EC scores) across root cell types in translatome and transcriptome

No. of cell types	Array element	Locus identifier	Annotation
4	260067_at	AT1G73780	Protease inhibitor/seed storage/lipid transfer protein (LTP) family protein
4	250108_at	AT5G15150	ATHB-3 (*Arabidopsis thaliana* HOMEOBOX 3); DNA binding/transcription factor
4	250322_at	AT5G12870	AtMYB46/MYB46 (myb domain protein 46); DNA binding/transcription factor
4	251009_at	AT5G02640	Similar to unknown protein [*Arabidopsis thaliana*] (TAIR:AT3G46300.1); similar to hyp. protein [*Vitis vinifera*] (GB:CAN66779·1)
4	260173_at	AT1G71930	VND7 (VASCULAR RELATED NAC-DOMAIN PROTEIN 7); transcription factor
4	253076_at	AT4G36160	ANAC076/VND2 (VASCULAR-RELATED NAC-DOMAIN 2); transcription factor
4	267613_at	AT2G26700	Protein kinase family protein
4	253120_at	AT4G35790	ATPLDDELTA (*Arabidopsis thaliana* phospholipase D delta); phospholipase D
4	266342_at	AT2G01540	C2 domain-containing protein
4	260468_at	AT1G11100	SNF2 domain-containing protein/helicase domain-containing protein/zinc finger protein-related
5	255637_at	AT4G00750	Dehydration-responsive family protein
5	260432_at	AT1G68150	WRKY9 (WRKY DNA-binding protein 9); transcription factor

In total, ten genes could be identified using the scenario considering the identical promoters and two genes considering the common promoters.

Remarkably, the majority of the identified 12 genes are transcription factors, or contain predicted DNA binding protein domains, e.g. *ATHB-3*, *MYB46*, *VND7* and *WRKY9*. Several of the genes are associated with key regulatory roles in roots, either for developmental or for response processes. For instance, *WRKY9* is involved in mediating cell responses to nutrient deprivation (Shin and Schachtman, 2004; Shin *et al*., 2005), and the transcription factor *MYB46* has a prominent role in the developmental programme of secondary wall biosynthesis (Zhong *et al*., 2007, 2013). In addition, *VND7* has been characterized as transcriptional master switches for plant meta- and protoxylem formation in arabidopsis (Kubo *et al*., 2005). Thus, the enrichment analysis of these genes suggests that certain key genes for root development maintain a direct relationship between expression and translation (Table 3).

**Table 3.** GSEA of genes displaying conserved expression/translation levels (PCC) and co-expression/co-translation relationships (EC scores) in the identical promoter dataset

GO term	Term description	No. of genes	*P*
GO:0006355	Regulation of transcription, DNA-dependent	4	<0·01
GO:0009741	Response to brassinosteroid stimulus	2	<0·01
GO:0010089	Xylem development	2	<0·01
GO:0010413	Glucuronoxylan metabolic process	2	<0·01
GO:0045492	Xylan biosynthetic process	2	<0·01
GO:0045893	Positive regulation of transcription, DNA-dependent	2	<0·01

Note that the corresponding two genes from the common promoter dataset did not result in a significant enrichment of GO-BP terms (cf. Table 2).

### Root cell type similarity based on transcriptome and translatome

**Fig. 8.**
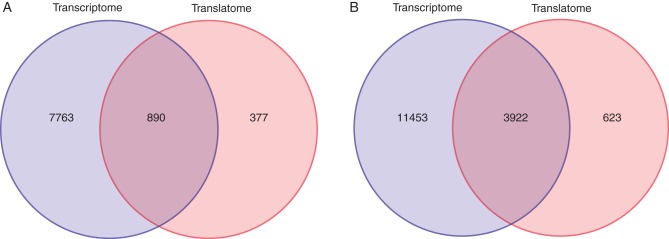
Visualization of the set of differentially expressed (DE) genes across the transcriptome and translatome of the identical (A) and common (B) promoter datasets. Differential expression was assessed by ANOVA at an FDR rate of 5 %. The numbers in the Venn diagram correspond to the number of DE genes found in each system level and the intersection thereof.

To complement the gene ‘centric’ analysis of (un-)coupled expression and translation, a cell type ‘centric’ analysis may reveal common themes among root cell types. Here, we attempted to elucidate whether transcriptional and/or translational patterns (termed themes) may be conserved across multiple cell types. To characterize a particular cell type, we first identified genes that showed differential expression and translation across the datasets. These estimates were derived for both the ‘identical’ and the ‘common’ promoter sets using ANOVA [FDR of 5 % by Benjamini–Hochberg (BH) multiple testing correction]. For the ‘identical’ promoters, a set of 890 genes displayed both differential expression and translation across the cell types (Fig. 8A). Moreover, for the five ‘common’ promoters, a set of 3923 genes showed differential expression and translation across the cell types (Fig. 8B). Considering expression and translation separately, we found that most genes exhibit differential expression across the cell types: ∼38 % (‘identical’ promoters) and 67 % (‘common’ promoters), while only ∼5 % (‘identical’ promoters) and ∼20 % (‘common’ promoters) of all genes display differential translation. Hierarchical clustering of the genes exhibiting differential expression on both levels, i.e. the 890 and 3923 genes above, revealed divergent patterns in cell-type-specificity (Supplementary Data Fig. S5).

**Fig. 9.**
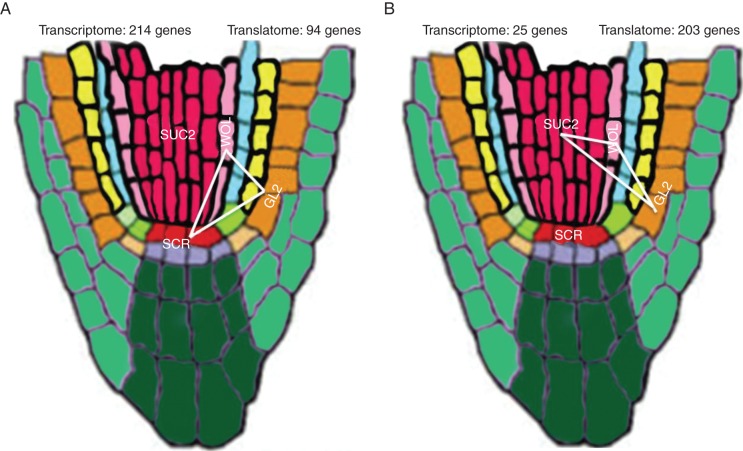
Two most commonly occurring network motifs for the transcriptome and translatome. A series of Tukey HSD tests were used on the common DE genes from the identical promoter dataset (890 genes) to detect significant pairwise cell type differences across the transcriptome and translatome. Network motifs were constructed using cell types as nodes and cell type similarity indicated by a red edge. Two network motifs, namely motif 1 and motif 2 in this figure, are represented by 279 and 214 unique genes, respectively.

**Fig. 10.**
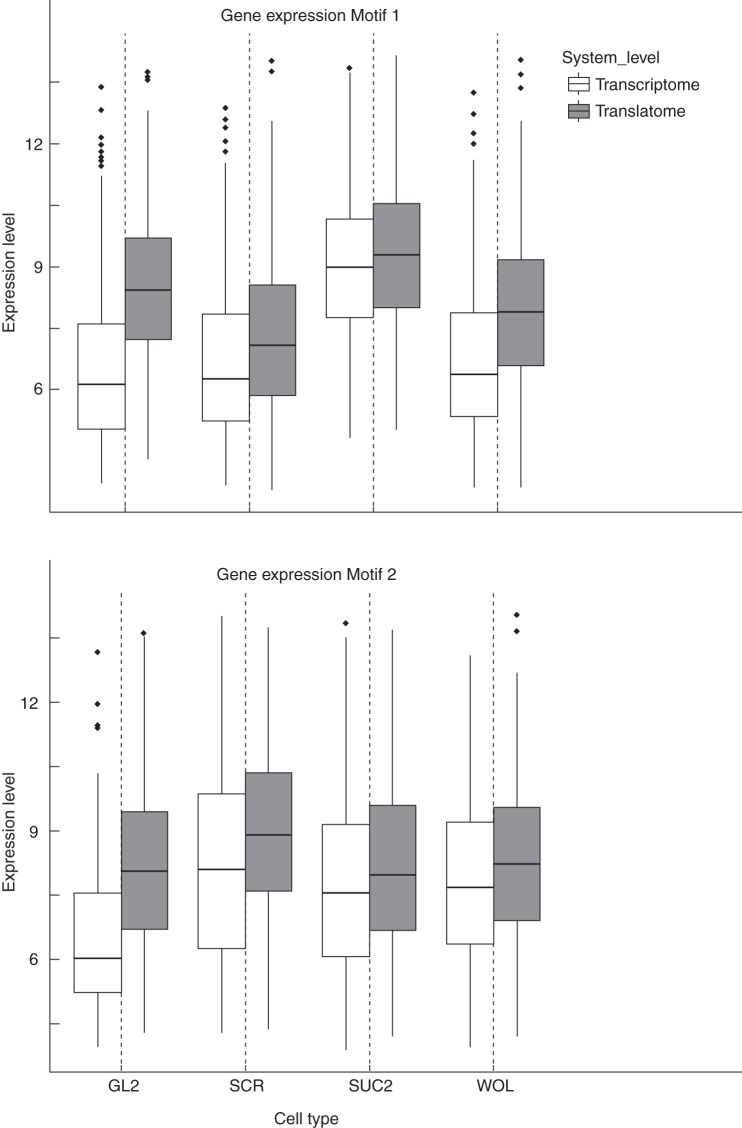
Boxplots of expression levels separated by promoters individually for translatome and transcriptome for network motif 1 (upper panel) and motif 2 (lower panel). In motif 1, 279 of 890 DE genes exhibit this characteristic cell-type-specific expression pattern. Furthermore, 214 genes correspond to motif 2.

We conducted a series of Tukey HSD tests (*P* < 0·05) for all genes displaying differential transcription and translation to further derive which cell-type-specific expression and translation levels differed significantly. Performed for each gene, the Tukey HSD post-hoc test allowed us to determine for which pair-wise cell type comparisons there is a significant difference in cell type expression/translation levels (Supplementary Data Fig. S6). When considering the four ‘identical’ promoters (representing cell types; Table 1) in the Tukey HSD test, we obtained a characteristic pattern of six pairwise cell type comparisons encoded for by a binary matrix (0 and 1). Then, for any given pair of cell types and system level, a significant difference in cell type expression or translation profile is assigned the value 1, and a similar expression profile is assigned a value of 0. We displayed the results as networks, here referred to as network *motifs*, to show patterns of similar gene levels. In these small networks, the four cell types (‘identical’ promoter dataset) correspond to nodes, and similarity between two cell types (=0) is indicated by an edge. From the 64 possible configurations of those networks (cf. Materials and Methods), we found that only five network motifs for the translatome and nine for the transcriptome occur more often than expected by chance (Supplementary Data Fig. S7). The significance of these motifs was tested empirically by permuting the results from the Tukey HSD test for each gene (i.e. shuffling the 0 and 1 values) and comparing the observed occurrence counts of genes for a particular motif with those obtained randomly over *n* = 1000 permutations (Supplementary Data Table S6). Furthermore, of all significantly occurring motifs only two are represented by a high number of genes [279 and 214 unique genes, motif 1 (Fig. 9A) and motif 2 (Fig. 9B), respectively] of the 890 differentially expressed genes. Both these motifs contain one isolated node, i.e. a cell type that is not connected by edges to any other cell types, indicating that this cell type is dissimilar based on the gene's expression or translation profile. Moreover, in both the motifs, the remaining three cell types are fully connected by edges, indicating similar behaviour of the relative levels of expression/translation of the genes included in the motif. The first motif (motif 1) shows that the majority of genes have dissimilar expression pattern in phloem companion cells (*SUC2*) as compared with the other three cell types (*WOL*, *SCR* and *GL2*). In the second motif (motif 2), the root quiescent centre (*SCR*) displays deviating expression patterns. Looking more closely at these differences shows that the major driving force behind the deviation in the phloem companion cells can be attributed to the transcriptomic datasets (214 differentially expressed genes), while the deviation of the quiescent root centre is largely due to differences in the translatome profiles (203 differentially expressed genes). An enrichment analysis using GO-BP terms associated with the genes in the first motif (214 genes) reveals mainly transport processes (general and transmembrane), as well as responses to sugar stimuli (glucose, sucrose and fructose) to be overrepresented (Supplementary Data Table S7). These data are in agreement with a major function of phloem companion cells in sugar transport (Stadler *et al*., 1995; Oparka and Turgeon, 1999; Williams *et al*., 2000). When we looked at the relative expression levels of the genes associated with this motif (again 214; Fig. 10) we found that the transcript levels were elevated. This is consistent with a role of the gene products in the function of these cells.

By contrast, genes associated with the second motif, i.e. where the root quiescent centre showed dissimilar expression patterns, were enriched for cell wall modification, xylan biosynthetic process and root hair cell differentiation/elongation. More importantly, the GO-BP terms oxidative stress, oxidation–reduction processes and auxin polar transport were also enriched. The quiescent centre cells typically accumulate high auxin levels that serve as a distal organizer (Sabatini *et al*., 1999). This is accompanied by the overproduction of reactive oxygen species. This is mediated by high levels of activity of ascorbate oxidase that cause reduction in the reduced form of ascorbic acid and glutathione and, simultaneously, an increase in the content of reactive oxygen species in the root quiescent centre cells (Ivanov, 2007; Tyburski *et al*., 2010). Oxidative stress represses proliferation of these cells, thus maintaining the cells in a quiescent state (Jiang *et al*., 2003). Expression levels of genes corresponding to this second motif are shown in Fig. 10, which indicates a relatively higher degree of translation in the root quiescent centre.

## DISCUSSION

A long-standing question in cellular biology is how well the transcriptome is coupled to the proteome (Zanetti *et al*., 2005). Profiling of mRNAs associated with polysomes can give a rough estimate of a cell's or tissue's proteome. Hence, by comparing cell-type-specific levels of total and polysomal mRNA in a global context, one can derive to what extent expression and translation are coupled. Based on the efforts of Brady *et al*. (2007) and Mustroph *et al*. (2009) the arabidopsis root atlas allows us to analyse transcriptomic and translatomic data sets and to identify particular genes that show either a tight coupling, or an uncoupling, of expression and translation profiles over a collection of cell types. On the computational level, our study represents an extension to the analysis of Mustroph *et al*. (2009), who used their root and shoot data in combination with hypoxia conditions to identify DE genes at the single cell-, region- and organ-specific levels. Recently, Lin *et al*. (2014) have investigated the translatome of *in vivo*-grown pollen tubes from self-pollinated gynoecia of arabidopsis. By using a pollen-specific promoter, epitope-tagged polysomal–RNA complexes could be affinity purified to obtain mRNAs undergoing translation. The authors also employed joint (RMA-) normalization to compare the translatome data with publicly available transcriptomics datasets. Set theory and analysis of the differential behaviour of genes finally identified a group of genes important in *in vivo* pollen tube biology.

Furthermore, we examined the variance in expression and translation levels using CVs and tested the similarity of gene expression/translation patterns across the root cell types using PCC. The observed change in CVs (Fig. 3) and the presence of negative PCC values (Fig. 4) when globally comparing the translatome and transcriptome is similar to what was found by Tebaldi *et al*. (2012). Here, the authors concluded a general uncoupling of the translatome and transcriptome based on low correlations found using epidermal growth factor stimulation in mammalian HeLa cells. Uncoupling of transcriptome and translatome has also been documented in human and yeast cells in response to various stimuli and stresses (Mikulits *et al*., 2000; Grolleau *et al*., 2002). For example, yeast exposed to different stresses, such as amino acid depletion and fusel alcohol addition, show distinct translational profiles (Smirnova *et al*., 2005), suggesting there is a distinct role of translational regulation for rapid responses in cells to environmental stress. However, by further focusing the analysis to the level of individual genes, our results also revealed groups of genes displaying coupled transcription/translation involved in processes such as stress responses (e.g. wounding, bacteria, nitrogen starvation and osmotic stress). These findings are, on the other hand, in agreement with the study in yeast by Halbeisen and Gerber (2009), who found relatively high overall PCC values of 0·75–0·81 of the overall genomic (fold-) changes in expression upon different conditions of cellular stress, such as osmotic stress between transcriptome and translatome.

In our study, genes that show correlated transcription and translation are enriched in cell-wall-related processes, which is in agreement with co-expression approaches that have successfully been undertaken for secondary wall synthesis (Persson *et al*., 2005). Here, many secondary wall genes are transcriptionally and functionally coordinated, which implies that the translation also would be coordinated with the transcription (Mutwil *et al*., 2009; Ruprecht and Persson, 2012). While these processes appear to be coupled, most of the genes displayed uncoupled transcription and translation in the cell types considered in our analysis.

In addition, a high degree of uncoupling between transcription and translation was observed when investigating correlations in co-expression and co-translation relationships. Here, over 12 000 genes displayed altered co-expression patterns in the eight ‘common’ promoter datasets (Fig. 2). This may reflect a re-wiring of co-regulation of genes during translation compared with transcription. In addition, cell-type-specific mRNA abundance appeared different on the two levels with 11 453 genes differentially expressed exclusively in the transcriptome (Fig. 8B). Notably, large proportions of genes displaying conserved co-expression/co-translation neighbourhoods are transcription factors or are putatively involved in regulation of transcription.

Note that bootstrapping procedures were carried out to ensure the robustness of our analyses. The benefits of this approach are two-fold: the random PCC and EC scores account for, first, sample size and, second, differences in cell type promoter specificity and the presence of multiple promoters targeting the same cell type in the case of the ‘common’ promoter dataset. As a consequence, one can robustly classify genes whose total and polysomal-associated mRNA levels are coupled (high PCC and *Z-*score of ≥1·96) as well as genes that display an uncoupling of both mRNA levels (low PCC and *Z-*score of ≤–1·96). Accordingly, we employed the same statistical framework to confirm the similarity of co-expression and co-translation neighbourhoods in the network analysis to ensure robustness. Nevertheless, the observed effects must be carefully interpreted given that the datasets originate from different labs and, moreover, rely on different extraction procedures. Here, the identified coupled attributes of gene expression on the transcriptional and translational level are therefore remarkable. Moreover, many of our observations are in close agreement with well-established characteristics of root cell type function and development.

One of the limitations of our study is the available selection of promoters, i.e. cell types, for the datasets. Clearly, in the case of the correlation analyses of transcription and translation of the individual genes, a greater sample size would have been desirable. Also, in the case of the ‘common’ five cell types, artefacts may arise due to slight variation in promoter strength and specificity across the cell types. Therefore, it is impossible to rule out deviations in transcription and translation based on promoter patterns. Nevertheless, we found correlation between transcription and translation for genes that we anticipated, such as for the secondary wall genes discussed above. These results are reassuring, and may provide a foundation for future efforts in this area. We propose that using more cell-type-specific promoters and performing the transcript and translatome analyses in one lab using the same methods will generate a robust and interesting data series that may be used to improve our results. Such datasets would be of immense interest to understand coupled and un-coupled gene regulation in arabidopsis roots.

## SUPPLEMENTARY DATA

Supplementary data are available online at www.aob.oxfordjournals.org and consist of the following. Gene List: details of all genes identified at any step of the analysis. Promoter Sequences: We have provided the promoter sequences of both the transcriptome and translatome dataset. Fig. S1: normalization of the CEL files. Fig. S2: histogram displaying the effects of joint and individual RMA normalization of the raw data (CEL files) from transcriptome and translatome. Fig. S3: correlation between the replicates. Fig. S4: degree of distribution and edge betweenness of the co-expression and co-translation network. Fig. S5: relative gene expression levels for the 890 genes for identical promoters displaying both differential expression and translation and 3922 genes with significant differences across common promoters in transcriptome and translatome. Fig. S6: conversion of pairwise differences in cell-type-specific expression levels derived by Tukey's HSD test, to cell type similarity networks. Fig. S7: all possible motif occurrences across the identical promoter data of the transcriptome and translatome. Table S1: list of available cell types and their corresponding promoters in the transcriptome and translatome dataset. Table S2: enriched GO terms of coupled/uncoupled gene expression patters on translatome and transcriptome for the identical promoter data. Table S3: enriched GO terms of coupled/uncoupled gene expression patterns on translatome and transcriptome for the common promoters. Table S4: GSEA of GO-BP terms for genes showing altered expression conservation for identical promoter data. Table S5: GSEA of GO-BP terms for altered expression conservation genes for the common promoters. Table S6: all possible motif occurrences across the identical promoters of transcriptome and translatome. The observed motifs are depicted in the order mentioned in the third column header. A characteristic pattern of the pairwise differences is represented by 1 (significant mean difference of expression values) and 0 (no significant mean difference of expression values). Table S7: enriched GO terms of genes with characteristic cell-type-specific gene expression patters found for motif 1 and motif 2.

Supplementary Data

## References

[MCU151C1] Arakawa K, Tomita M (2013). Merging multiple omics datasets in silico: statistical analyses and data interpretation. Methods in Molecular Biology.

[MCU151C2] Ashburner M, Ball CA, Blake JA (2000). Gene ontology: tool for the unification of biology. The Gene Ontology Consortium. . Nature Genetics.

[MCU151C3] Baerenfaller K, Grossmann J, Grobei MA (2008). Genome-scale proteomics reveals *Arabidopsis thaliana* gene models and proteome dynamics. Science.

[MCU151C4] Baginsky S, Hennig L, Zimmermann P, Gruissem W (2010). Gene expression analysis, proteomics, and network discovery. Plant Physiology.

[MCU151C5] Barrett T, Edgar R (2006). Gene expression omnibus: microarray data storage, submission, retrieval, and analysis. Methods in Enzymology.

[MCU151C6] Benfey PN, Bennett M, Schiefelbein J (2010). Getting to the root of plant biology: impact of the arabidopsis genome sequence on root research. The Plant Journal.

[MCU151C7] Benjamini Y, Hochberg Y (1995). Controlling the false discovery rate: a practical and powerful approach to multiple testing. Journal of the Royal Statistical Society Series B (Methodological).

[MCU151C8] Bevan M, Walsh S (2005). The arabidopsis genome: a foundation for plant research. Genome Research.

[MCU151C9] Birnbaum K, Shasha DE, Wang JY (2003). A gene expression map of the arabidopsis root. Science.

[MCU151C10] Birnbaum K, Jung JW, Wang JY (2005). Cell type-specific expression profiling in plants via cell sorting of protoplasts from fluorescent reporter lines. Nature Methods.

[MCU151C11] Brady SM, Orlando DA, Lee J-Y (2007). A high-resolution root spatiotemporal map reveals dominant expression patterns. Science.

[MCU151C12] Brandt SP (2005). Microgenomics: gene expression analysis at the tissue-specific and single-cell levels. Journal of Experimental Botany.

[MCU151C13] Brink-Jensen K, Bak S, Jørgensen K, Ekstrøm CT (2013). Integrative analysis of metabolomics and transcriptomics data: a unified model framework to identify underlying system pathways. PloS one.

[MCU151C14] Butte AJ, Kohane IS, Parmigiani G, Garrett ES, Irizarry RA, Zeger SL (2003). Relevance networks: a first step toward finding genetic regulatory networks within microarray data. Statistics for biology and health. The analysis of gene expression data.

[MCU151C15] Chartrand G (1985). Introductory graph theory.

[MCU151C16] Dutilh BE, Huynen MA, Snel B (2006). A global definition of expression context is conserved between orthologs, but does not correlate with sequence conservation. BMC Genomics.

[MCU151C17] Edwards JW, Coruzzi GM (1990). Cell-specific gene expression in plants. Annual Review of Genetics.

[MCU151C18] Grolleau A, Bowman J, Pradet-Balade B (2002). Global and specific translational control by rapamycin in T cells uncovered by microarrays and proteomics. The Journal of Biological Chemistry.

[MCU151C19] Hack CJ (2004). Integrated transcriptome and proteome data: the challenges ahead. Briefings in Functional Genomics & Proteomics.

[MCU151C20] Halbeisen RE, Gerber AP (2009). Stress-dependent coordination of transcriptome and translatome in yeast. PLoS biology.

[MCU151C21] Hamilton JP, Robin Buell C (2012). Advances in plant genome sequencing. The Plant Journal.

[MCU151C22] Hannah MA, Caldana C, Steinhauser D, Balbo I, Fernie AR, Willmitzer L (2010). Combined transcript and metabolite profiling of arabidopsis grown under widely variant growth conditions facilitates the identification of novel metabolite-mediated regulation of gene expression. Plant Physiology.

[MCU151C23] Huttenhower C, Hibbs M, Myers C, Troyanskaya OG (2006). A scalable method for integration and functional analysis of multiple microarray datasets. Bioinformatics.

[MCU151C24] Irizarry RA, Hobbs B, Collin F (2003). Exploration, normalization, and summaries of high density oligonucleotide array probe level data. Biostatistics.

[MCU151C25] Ivanov VB (2007). Oxidative stress and formation and maintenance of root stem cells. Biochemistry. Biokhimiia⌢.

[MCU151C26] Iyer-Pascuzzi AS, Benfey PN (2010). Fluorescence-activated cell sorting in plant developmental biology. Methods in Molecular Biology.

[MCU151C27] Jiang K, Meng YL, Feldman LJ (2003). Quiescent center formation in maize roots is associated with an auxin-regulated oxidizing environment. Development.

[MCU151C28] Joyce AR, Palsson BØ (2006). The model organism as a system: integrating ‘omics’ data sets. Nature Reviews. Molecular Cell Biology.

[MCU151C29] Kerr MK, Martin M, Churchill GA (2000). Analysis of variance for gene expression microarray data. Journal of Computational Biology.

[MCU151C30] Kleffmann T, Russenberger D, von Zychlinski A (2004). The *Arabidopsis thaliana* chloroplast proteome reveals pathway abundance and novel protein functions. Current Biology.

[MCU151C31] Klie S, Nikoloski Z (2012). The choice between MapMan and Gene Ontology for automated gene function prediction in plant science. Frontiers in Bioinformatics and Computational Biology.

[MCU151C32] Kubo M, Udagawa M, Nishikubo N (2005). Transcription switches for protoxylem and metaxylem vessel formation. Genes & Development.

[MCU151C33] Lee J-Y, Colinas J, Wang JY, Mace D, Ohler U, Benfey PN (2006). Transcriptional and posttranscriptional regulation of transcription factor expression in arabidopsis roots. Proceedings of the National Academy of Sciences of the United States of America.

[MCU151C34] Liepman AH, Wightman R, Geshi N, Turner SR, Scheller HV (2010). Arabidopsis – a powerful model system for plant cell wall research. The Plant Journal.

[MCU151C35] Lin S-Y, Chen P-W, Chuang M-H, Juntawong P, Bailey-Serres J, Jauh G-Y (2014). Profiling of translatomes of *in vivo*–grown pollen tubes reveals genes with roles in micropylar guidance during pollination in arabidopsis. The Plant Cell Online.

[MCU151C36] Mikulits W, Pradet-Balade B, Habermann B, Beug H, Garcia-Sanz JA, Müllner EW (2000). Isolation of translationally controlled mRNAs by differential screening. The FASEB Journal.

[MCU151C37] Milioni D, Sado P-E, Stacey NJ, Roberts K, McCann MC (2002). Early gene expression associated with the commitment and differentiation of a plant tracheary element is revealed by cDNA-amplified fragment length polymorphism analysis. The Plant Cell.

[MCU151C38] Milo R, Shen-Orr S, Itzkovitz S, Kashtan N, Chklovskii D, Alon U (2002). Network motifs: simple building blocks of complex networks. Science.

[MCU151C39] Mochida K, Shinozaki K (2010). Genomics and bioinformatics resources for crop improvement. Plant and Cell Physiology.

[MCU151C40] Mustroph A, Zanetti ME, Jang CJH (2009). Profiling translatomes of discrete cell populations resolves altered cellular priorities during hypoxia in arabidopsis. Proceedings of the National Academy of Sciences of the United States of America.

[MCU151C41] Mutwil M, Ruprecht C, Giorgi FM, Bringmann M, Usadel B, Persson S (2009). Transcriptional wiring of cell wall-related genes in arabidopsis. Molecular Plant.

[MCU151C42] Nawy T, Lee J-Y, Colinas J (2005). Transcriptional profile of the arabidopsis root quiescent center. The Plant Cell.

[MCU151C43] Oparka KJ, Turgeon R (1999). Sieve elements and companion cells—traffic control centers of the phloem. The Plant Cell Online.

[MCU151C44] Osorio S, Alba R, Nikoloski Z, Kochevenko A, Fernie AR, Giovannoni JJ (2012). Integrative comparative analyses of transcript and metabolite profiles from pepper and tomato ripening and development stages uncovers species-specific patterns of network regulatory behavior. Plant Physiology.

[MCU151C45] Pan Z, Zeng Y, An J, Ye J, Xu Q, Deng X (2012). An integrative analysis of transcriptome and proteome provides new insights into carotenoid biosynthesis and regulation in sweet orange fruits. Journal of Proteomics.

[MCU151C46] Persson S, Wei H, Milne J, Page GP, Somerville CR (2005). Identification of genes required for cellulose synthesis by regression analysis of public microarray data sets. Proceedings of the National Academy of Sciences of the United States of America.

[MCU151C47] Petricka JJ, Schauer MA, Megraw M (2012). The protein expression landscape of the arabidopsis root. Proceedings of the National Academy of Sciences of the United States of America.

[MCU151C48] Rivals I, Personnaz L, Taing L, Potier M-C (2007). Enrichment or depletion of a GO category within a class of genes: which test?. Bioinformatics.

[MCU151C49] Ruprecht C, Persson S (2012). Co-expression of cell-wall related genes: new tools and insights. Frontiers in Plant Science.

[MCU151C50] Sabatini S, Beis D, Wolkenfelt H (1999). An auxin-dependent distal organizer of pattern and polarity in the arabidopsis root. Cell.

[MCU151C51] Sauer U, Heinemann M, Zamboni N (2007). Genetics. *Getting closer to the whole picture.* Science.

[MCU151C52] Schauer N, Semel Y, Roessner U (2006). Comprehensive metabolic profiling and phenotyping of interspecific introgression lines for tomato improvement. Nature Biotechnology.

[MCU151C53] Shen-Orr SS, Tibshirani R, Khatri P (2010). Cell type-specific gene expression differences in complex tissues. Nature Methods.

[MCU151C54] Shin R, Schachtman DP (2004). Hydrogen peroxide mediates plant root cell response to nutrient deprivation. Proceedings of the National Academy of Sciences of the United States of America.

[MCU151C55] Shin R, Berg RH, Schachtman DP (2005). Reactive oxygen species and root hairs in arabidopsis root response to nitrogen, phosphorus and potassium deficiency. Plant and Cell Physiology.

[MCU151C56] Smirnova JB, Selley JN, Sanchez-Cabo F (2005). Global gene expression profiling reveals widespread yet distinctive translational responses to different eukaryotic translation initiation factor 2B-targeting stress pathways. Molecular and Cellular Biology.

[MCU151C57] Sokal RR, Rohlf FJ (1995). Biometry: the principles and practice of statistics in biological research.

[MCU151C58] Stadler R, Brandner J, Schulz A, Gahrtz M, Sauer N (1995). Phloem loading by the PmSUC2 sucrose carrier from *Plantago major* occurs into companion cells. The Plant Cell Online.

[MCU151C59] Stigler SM (1989). Francis Galton's account of the invention of correlation. Statistical Science.

[MCU151C60] Subramanian A, Tamayo P, Mootha VK (2005). Gene set enrichment analysis: a knowledge-based approach for interpreting genome-wide expression profiles. Proceedings of the National Academy of Sciences of the United States of America.

[MCU151C61] Swanson-Wagner R, Briskine R, Schaefer R (2012). Reshaping of the maize transcriptome by domestication. Proceedings of the National Academy of Sciences of the United States of America.

[MCU151C62] Tebaldi T, Re A, Viero G (2012). Widespread uncoupling between transcriptome and translatome variations after a stimulus in mammalian cells. BMC Genomics.

[MCU151C63] Tohge T, Nishiyama Y, Hirai MY (2005). Functional genomics by integrated analysis of metabolome and transcriptome of arabidopsis plants over-expressing an MYB transcription factor. The Plant Journal.

[MCU151C64] Tukey JW, Braun HI (1994). The collected works of John W. Tukey. Vol. 8.

[MCU151C65] Tyburski J, Dunajska K, Tretyn A (2010). A role for redox factors in shaping root architecture under phosphorus deficiency. Plant Signaling & Behavior.

[MCU151C66] Ventoso I, Kochetov A, Montaner D, Dopazo J, Santoyo J (2012). Extensive translatome remodeling during ER stress response in mammalian cells. PloS ONE.

[MCU151C67] Vogel C, Marcotte EM (2012). Insights into the regulation of protein abundance from proteomic and transcriptomic analyses. Nature Reviews Genetics.

[MCU151C68] Wang Y, Jiao Y (2011). Advances in plant cell type-specific genome-wide studies of gene expression. Frontiers in Biology.

[MCU151C69] Wang H, Wang Q, Pape UJ, Shen B, Huang J, Wu B, Li X (2010). Systematic investigation of global coordination among mRNA and protein in cellular society. BMC Genomics.

[MCU151C70] Williams LE, Lemoine R, Sauer N (2000). Sugar transporters in higher plants – a diversity of roles and complex regulation. Trends in Plant Science.

[MCU151C71] Zanetti ME, Chang I-F, Gong F, Galbraith DW, Bailey-Serres J (2005). Immunopurification of polyribosomal complexes of arabidopsis for global analysis of gene expression. Plant Physiology.

[MCU151C72] Zhang W, Li F, Nie L (2010). Integrating multiple ‘omics’ analysis for microbial biology: application and methodologies. Microbiology.

[MCU151C73] Zhong R, Richardson EA, Ye Z-H (2007). The MYB46 transcription factor is a direct target of SND1 and regulates secondary wall biosynthesis in arabidopsis. The Plant Cell.

[MCU151C74] Zhong R, McCarthy RL, Haghighat M, Ye Z-H (2013). The poplar MYB master switches bind to the SMRE site and activate the secondary wall biosynthetic program during wood formation. PloS ONE.

