# Supplementary material for: Co-ordination and divergence of cell-specific transcription and translation of genes in arabidopsis root cells
Source: Ann Bot. 2014 Aug 22;114(6):1109–23. doi: 10.1093/aob/mcu151 (PMC4195562; doi:10.1093/aob/mcu151)
Supplement: Supplementary Data [file supp_114_6_1109__index.html]

Co-ordination and divergence of cell-specific transcription and translation of genes in arabidopsis root cells — Supplementary Data 

# Co-ordination and divergence of cell-specific transcription and translation of genes in arabidopsis root cells

## Supplementary Data

Supplementary Data

**Files in this Data Supplement:**

- Supplementary Data - Pdf file
- Supplementary Table - csv file
- Transcriptome dataset- Promoter sequences - txt file
