# Supplementary material for: Co-ordination and divergence of cell-specific transcription and translation of genes in arabidopsis root cells
Source: Ann Bot. 2014 Aug 22;114(6):1109–23. doi: 10.1093/aob/mcu151 (PMC4195562; doi:10.1093/aob/mcu151)
Supplement: Supplementary Data [file supp_mcu151_mcu151supp.pdf]

SUPPLEMENTARY DATA

FIGURE CAPTIONS

Figure S1: Normalization of the CEL files. RMA normalization of the CEL files from the transcriptome and translatome was done together. The transcriptome dataset has 19 promoters in total with replicates (53 CEL files in total) which are shown in grey and the translatome dataset has 10 promoters with replicates (22 CEL files in total) coloured in red

Figure S2: Histogram displaying the effects of joint (blue) and individual (pink) RMA normalization of the raw data (CEL files) from transcriptome and translatome. The frequency of gene-expression values does not differ strongly in both scenarios.

Figure S3: Correlation between the replicates. (A) The plot shows the correlation between the replicates of the promoters targeting transcriptome cell-types. (B) This shows the correlation between the replicates of the promoters targeting translatome cell-types. Also, the number of replicates differs considerably in case of both datasets.

Figure S4A: Degree distribution of the co-expression and co-translation network. This figure displays the distribution of the degree for the co-expression (left) and co-translation network (right) using the 4 identical promoters. Clearly, the difference of both network-topologies becomes visible. However, the degree of the 12 genes (indicated by small *red* bars at the x-axis) is not strongly confined to a particular range, i.e. low degree or high degree.

Figure S4B: Edge betweenness of the co-expression and co-translation network. This figure displays the distribution of the betweenness for the co-expression (left) and co-translation network (right) using the 4 identical promoters. In this case, both network-structures seam more similar, but again, the betweenness of the 12 genes (indicated by small *red* bars at the x-axis) is evenly spaced across the whole range of obtained betweenness values. Note, that similar observation hold true both for other network properties as well as for the common set of promoters.

Figure S5A: Relative gene expression levels for the 890 DEG with significant differences across identical promoters in transcriptome (left) and translatome (right). The identical promoters are shown on the x-axes and the common DE genes along the y-axes. Common DE genes show divergent patterns of cell specificity across the two system levels.

Figure S5B: Relative gene expression levels for the 3922 genes with significant differences across common promoters in transcriptome (left) and translatome (right). The common

promoters are shown on the x-axes and the common DE genes along the y-axes. Common DE genes show divergent patterns of cell specificity across the transcriptome and translatome.

Figure S6: Conversion of pairwise differences in cell-type-specific expression levels derived by Tukey's HSD test, to cell-type similarity networks. Gene 1 is overexpressed in phloem companion cells (SUC2), rendering this cell-type different from the remaining 3 cell-types (represented by no edge). In case of gene 2, there exists no significant difference in cell-type-specific expression levels. As a result, all cell-types are similar, further represented by edges being present.

Figure S7: All possible motif occurrences across the identical promoter data of the transcriptome and translatome. Out of all possible network motifs for the transcriptome and translatome, only nine for the transcriptome and five for the translatome occur more often than expected by chance. The "C" and "L" used in the figure indicate the transcriptome and translatome respectively and give the number of genes which coincide with each motif.

Figure S1

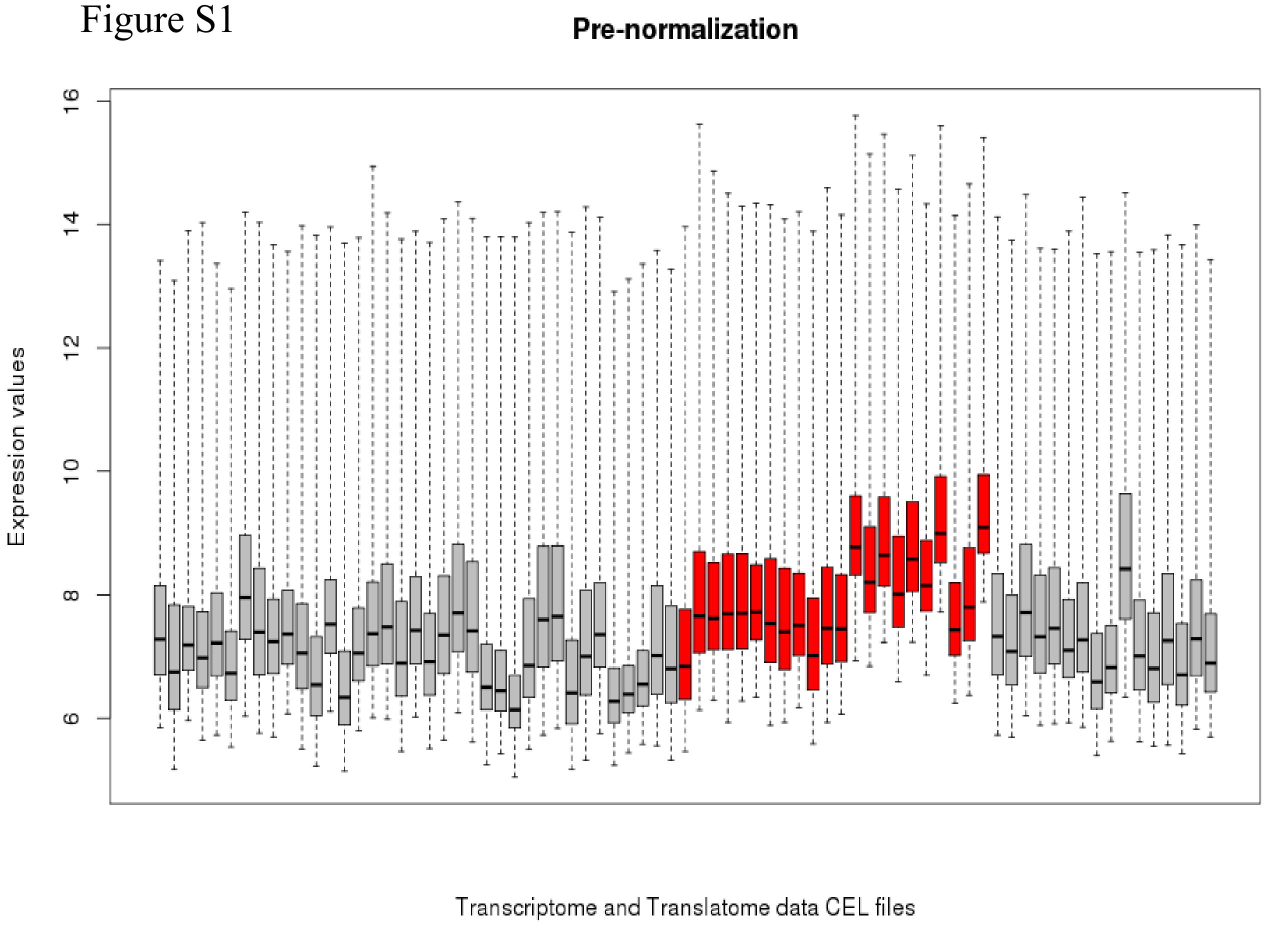
Pre-normalization
Expression values
6
8
10
12
14
16
Transcriptome and Translatome data CEL files

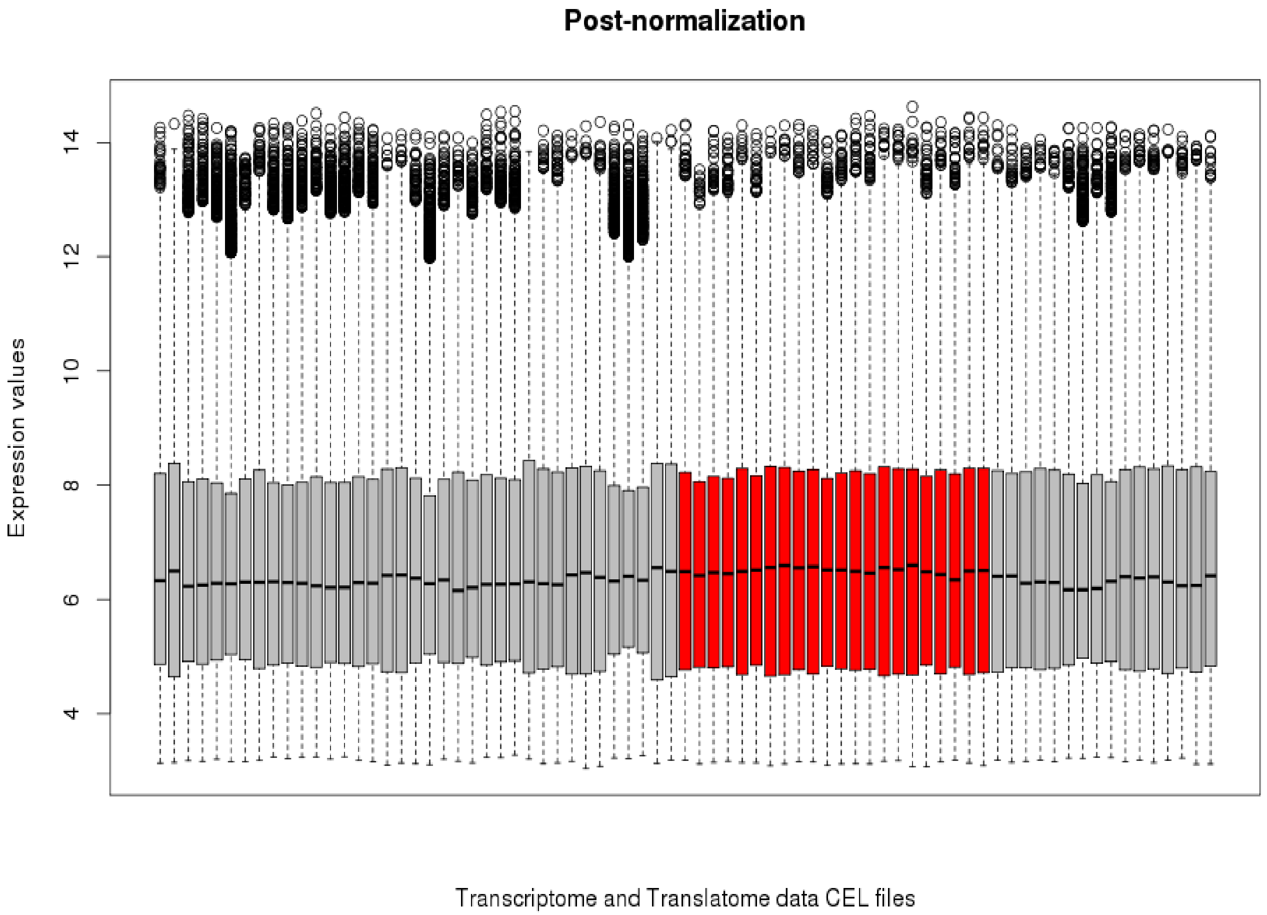
Post-normalization
Expression values
4
6
8
10
12
14
Transcriptome and Translatome data CEL files

Figure S2

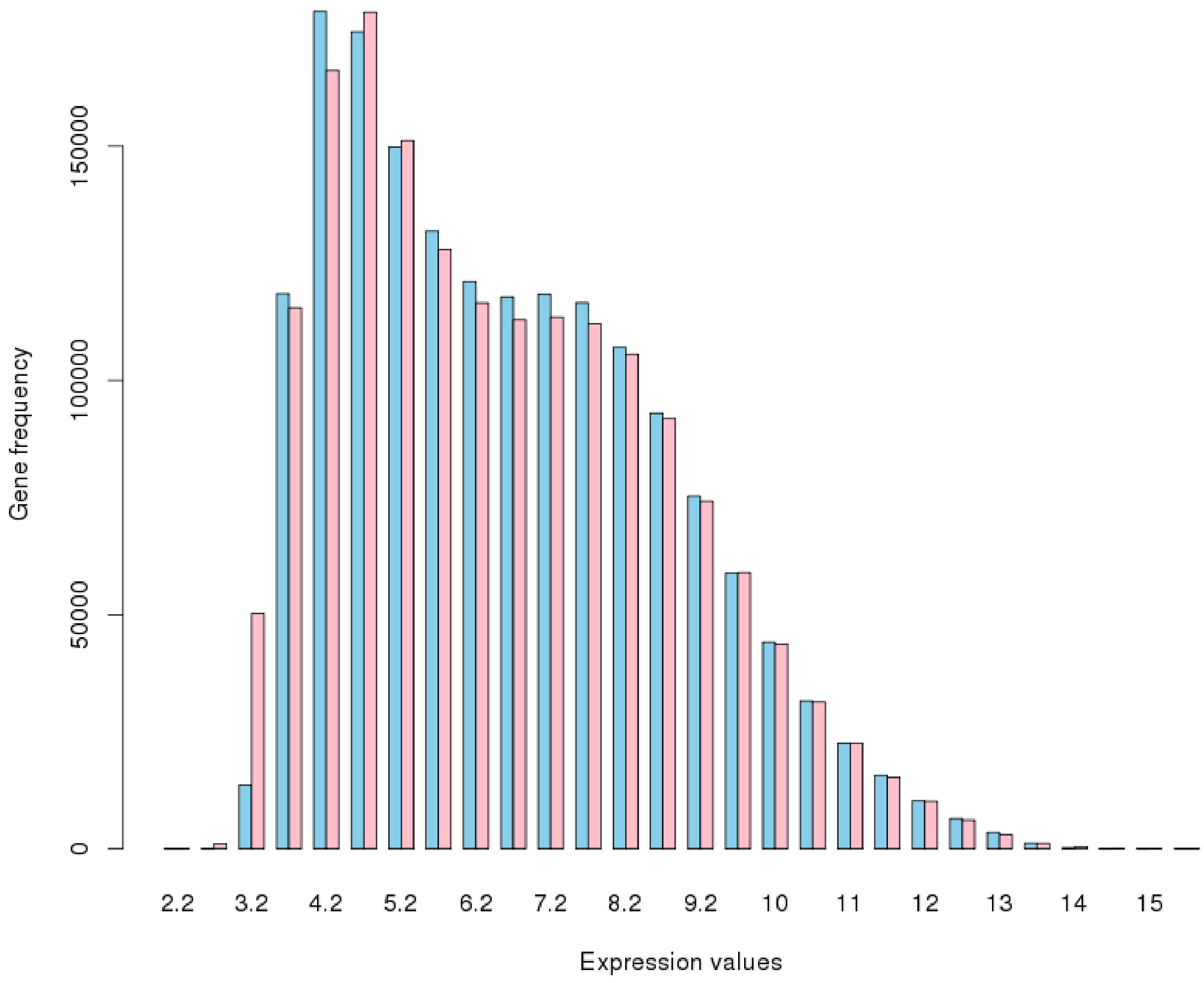

Gene frequency
0
50000
100000
150000
2.2
3.2
4.2
5.2
6.2
7.2
8.2
9.2
10
11
12
13
14
15
Expression values

Figure S3

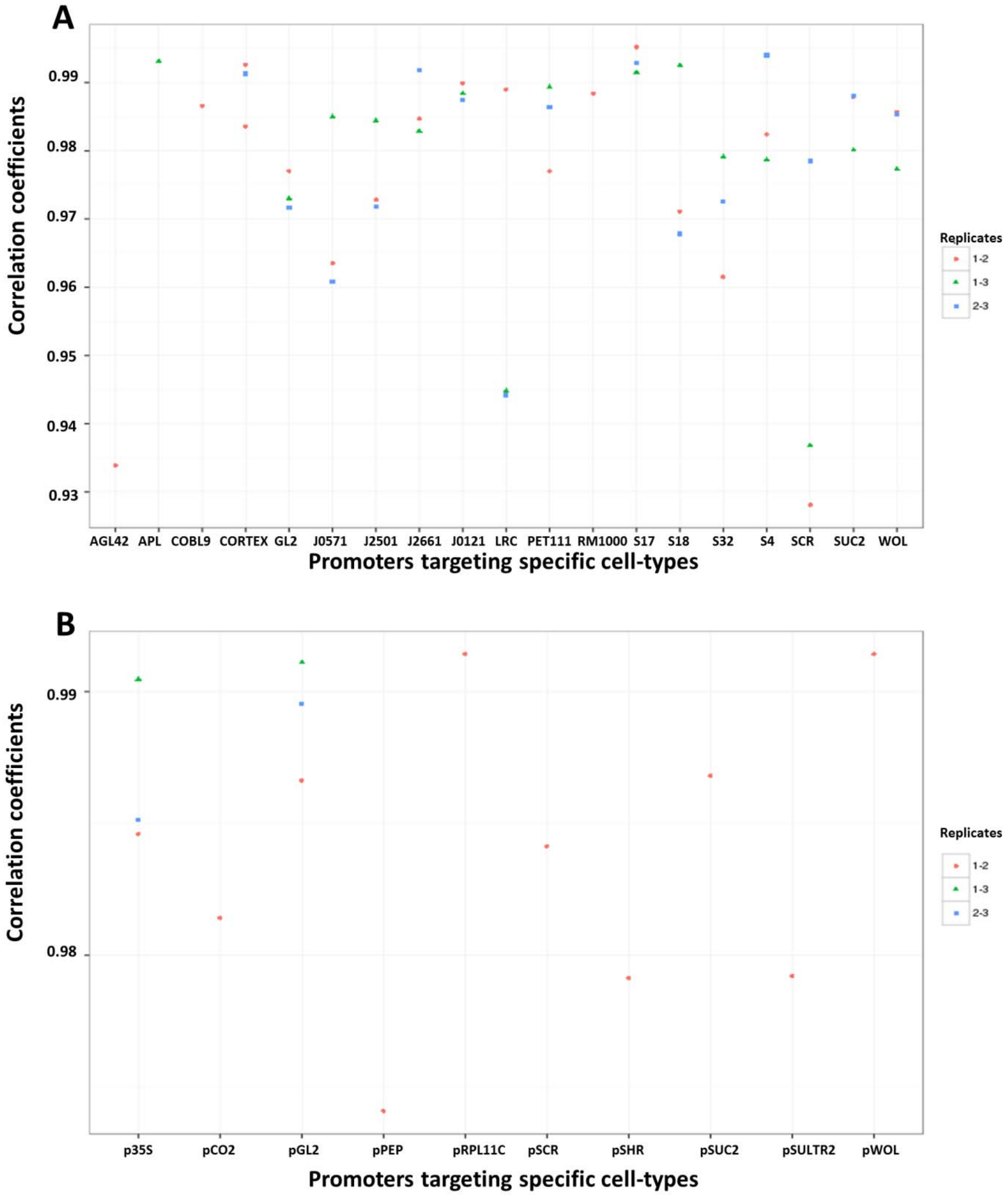
A
Correlation coefficients
0.99
0.98
0.97
0.96
0.95
0.94
0.93
AGL42 APL COBL9 CORTEX GL2 J0571 J2501 J2661 J0121 LRC PET111 RM1000 S17 S18 S32 S4 SCR SUC2 WOL
Promoters targeting specific cell-types
Replicates
1-2
1-3
2-3
B
Correlation coefficients
0.99
0.98
p35S pCO2 pGL2 pPEP pRPL11C pSCR pSHR pSUC2 pSULTR2 pWOL
Promoters targeting specific cell-types
Replicates
1-2
1-3
2-3

Figure S4A

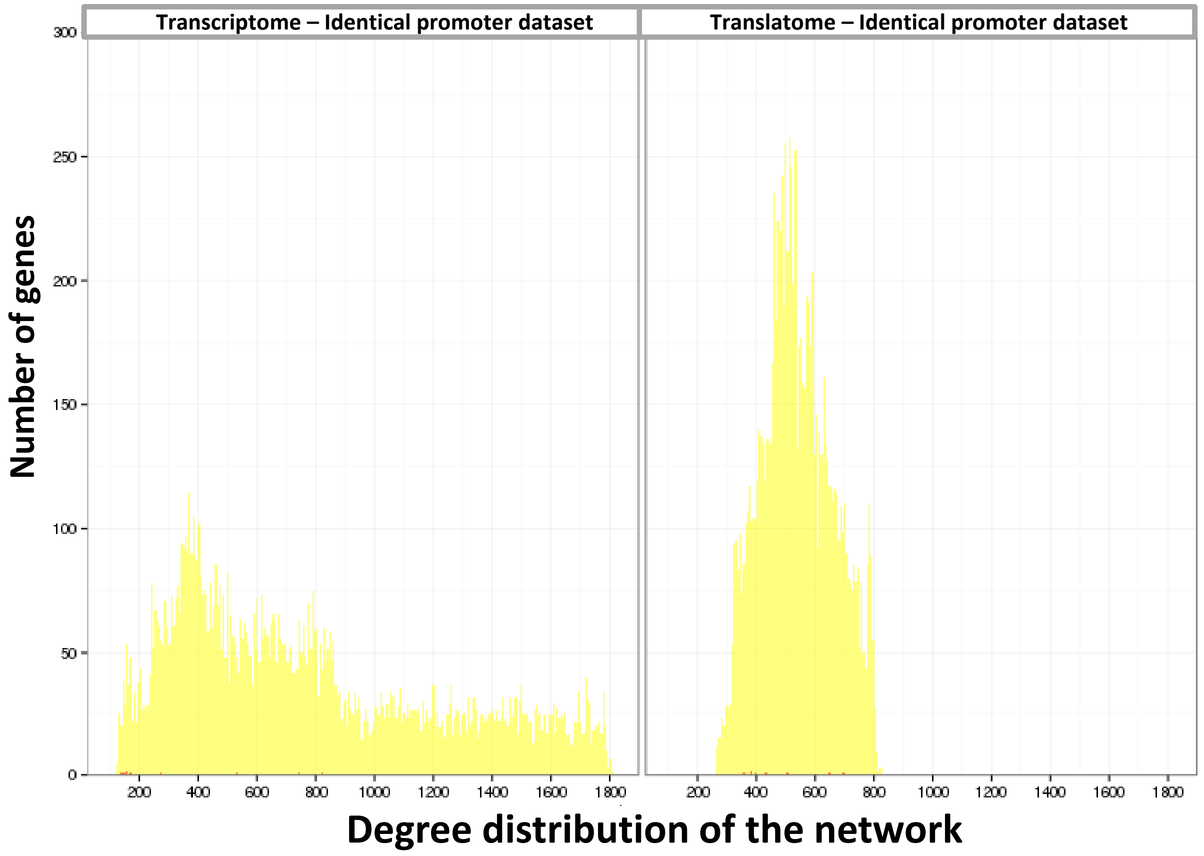

Figure S4B

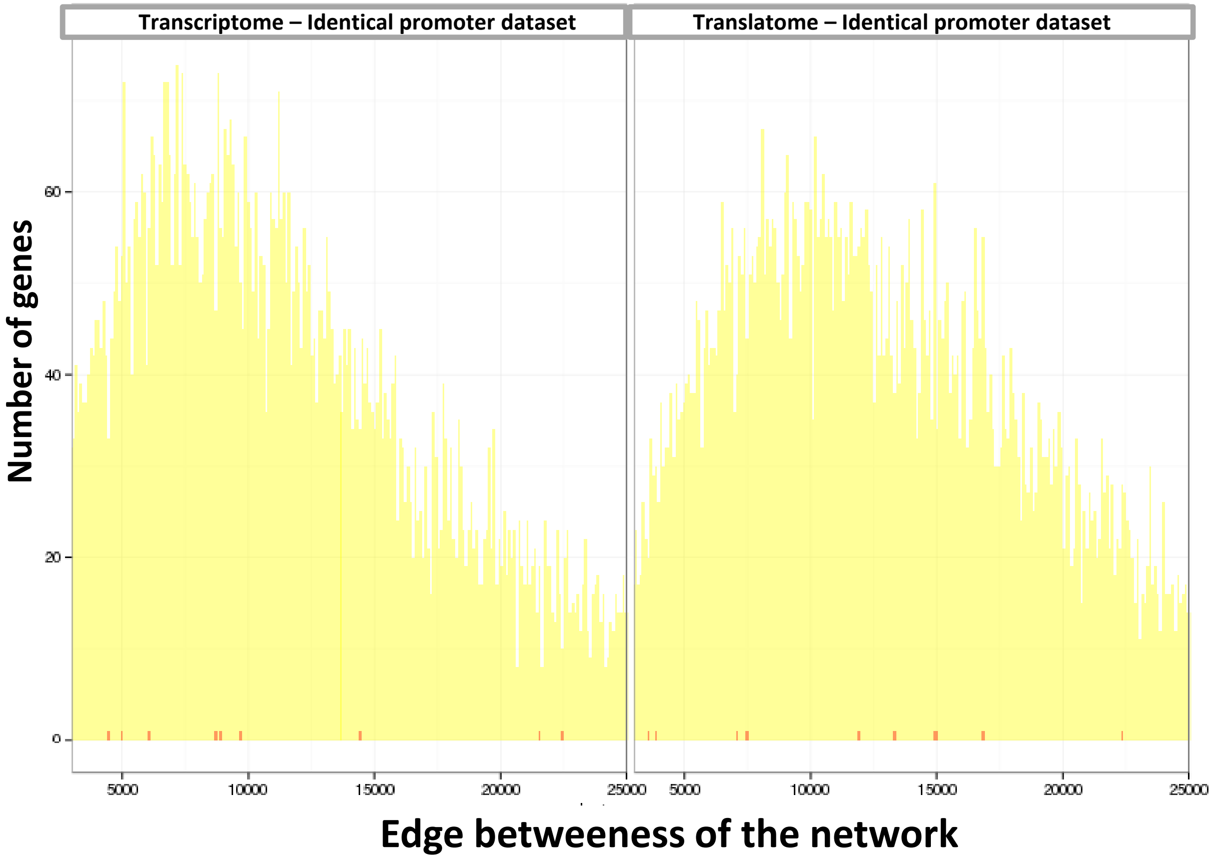

Figure S5A

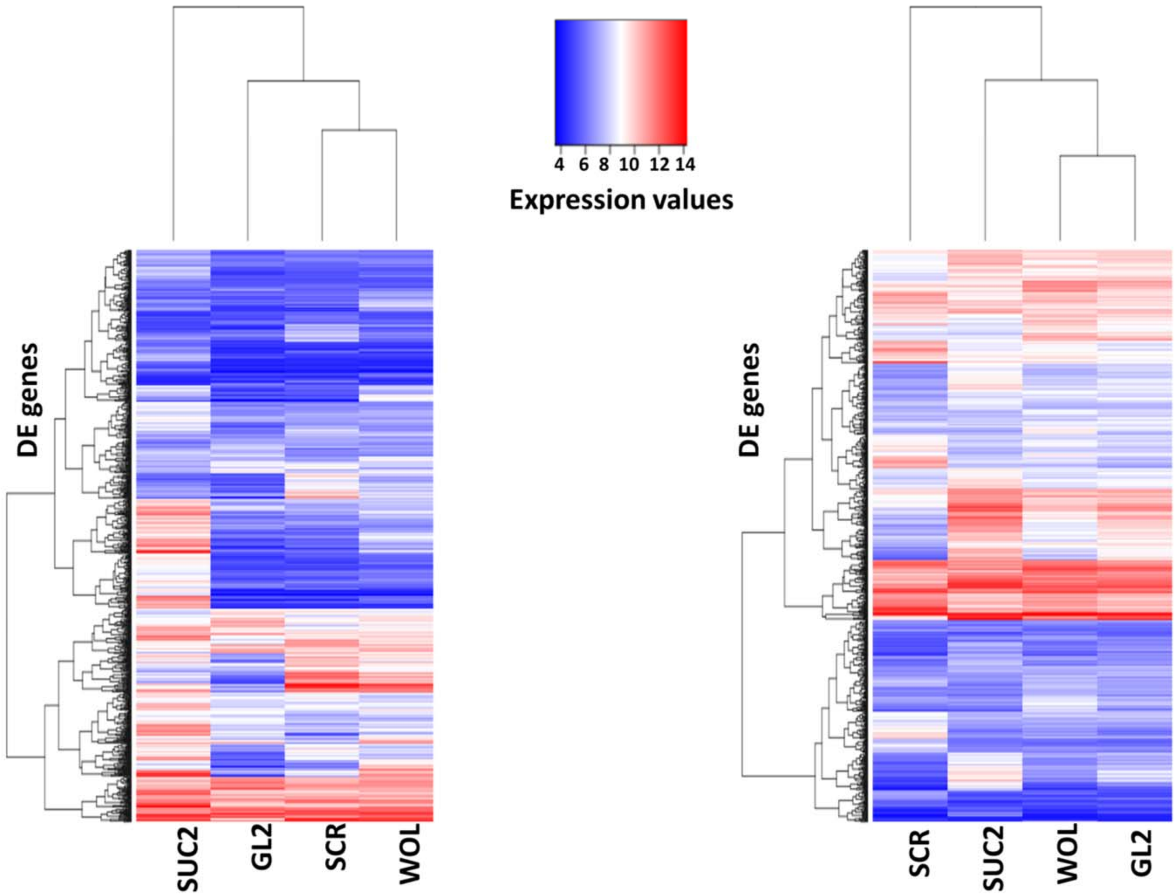

Figure S5B

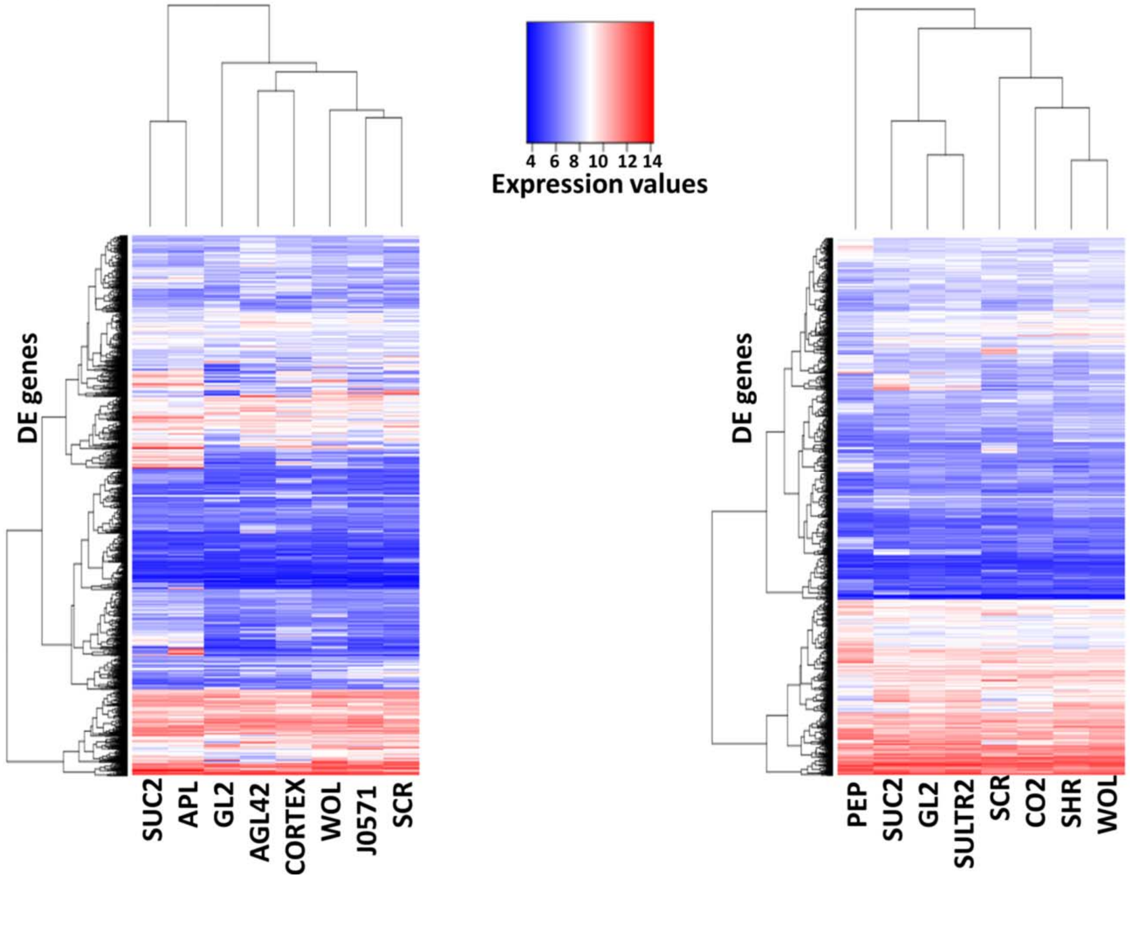

Figure S6

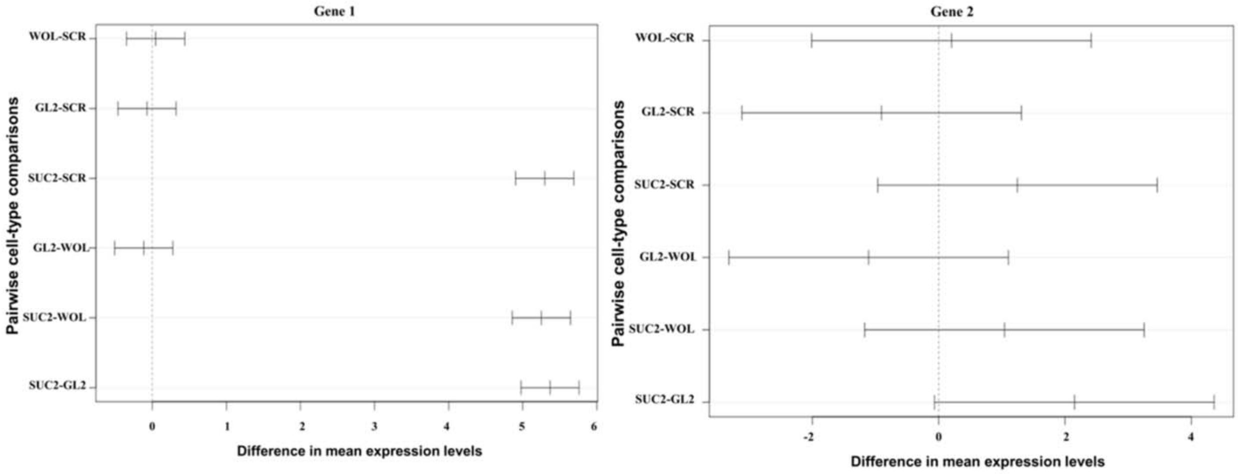

	WOL-SCR	GL2-SCR	SUC2-SCR	GL2-WOL	SUC2-WOL	SUC2-GL2
Gene 1	0	0	1	0	1	1
Gene 2	0	0	0	0	0	0

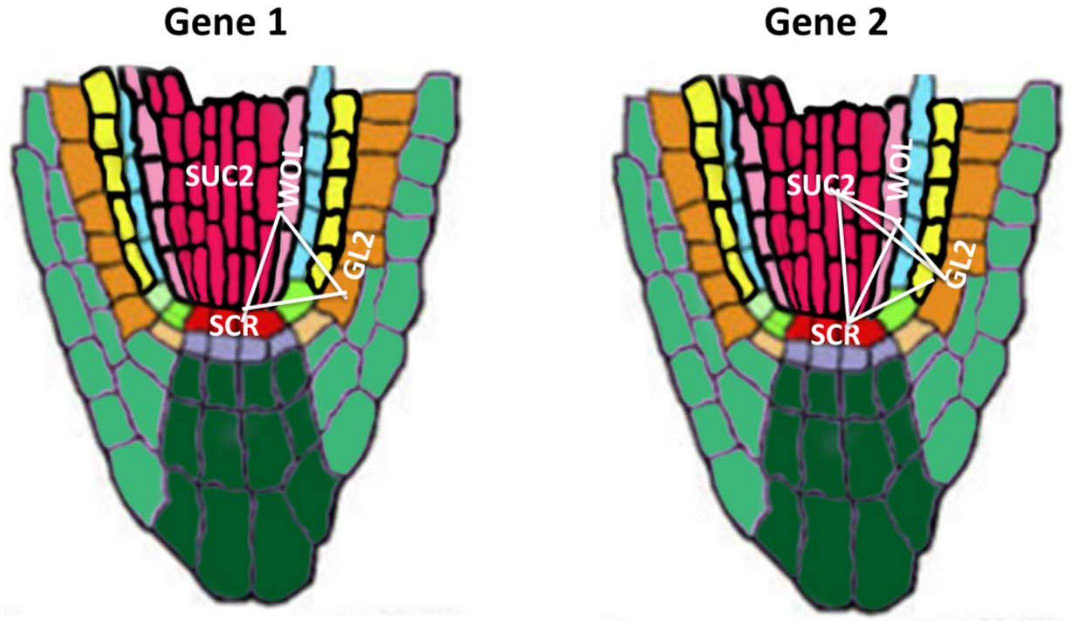

Figure S7

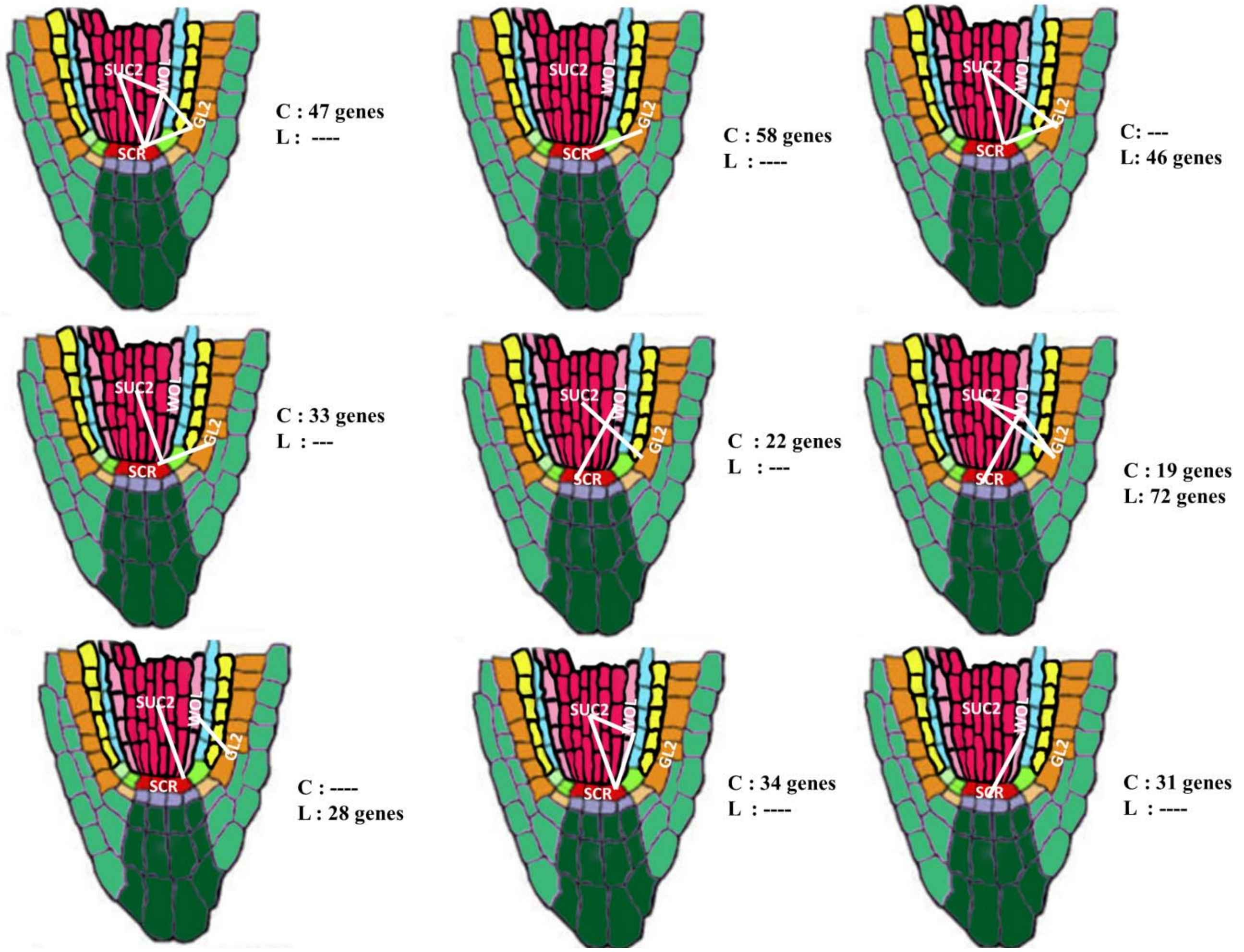
SUC2
WOL
GL2
SCR
C : 47 genes
L : ----
C : 58 genes
L : ----
C: ---
L: 46 genes
C : 33 genes
L : ---
C : 22 genes
L : ---
C : 19 genes
L: 72 genes
C : ----
L : 28 genes
C : 34 genes
L : ----
C : 31 genes
L : ----

SUPPLEMENTARY DATA

Table S1: List of available cell types and their corresponding promoters in the transcriptomeand translatome dataset.

Promoter	Targeting cell type	Dataset	Dataset source	Promoter source
AGL 42	Quiescent centre	Transcriptome	Nawy et al. 2005	Nawy et al.2005
PET 111	Columella tier 2	Transcriptome	Nawy et al. 2005	Nawy et al.2005
LRC	Lateral root cap	Transcriptome	Birnbaum et al.2003	Birnbaum et al.2003
GL2	Non-hair cells	Transcriptome	Birnbaum et al.2003	Birnbaum et al.2003
J0571	Ground endodermis+cortex+quiescent centre	Transcriptome	Birnbaum et al.2003	Birnbaum et al.2003
S17	Phloem pole pericycyle	Transcriptome	Brady, Orlando, et al. 2007	Lee et al., 2006
S32	Protophloem and Metaphloem	Transcriptome	Brady, Orlando, et al. 2007	Lee et al., 2006
COBL9	Isolated hair cells	Transcriptome	Brady, Orlando, et al. 2007	Brady, Song, et al. 2007
JO121	Xylem pole pericycle	Transcriptome	Brady, Orlando, et al. 2007	Laplaze et al. 2005
S4	Isolated Protoxylem and Metaphloem	Transcriptome	Brady, Orlando, et al. 2007	Lee et al., 2006
WOL	Stele	Transcriptome	Birnbaum et al.2003	Birnbaum et al.2003
SCR	Quiescent centre	Transcriptome	Birnbaum et al.2003	Birnbaum et al.2003
SUC2	Phloem companion cells	Transcriptome	Brady, Orlando, et al. 2007	Imlau et al. 1999
J2501	Pericycle,protoxylem,metaxylem	Transcriptome	Brady, Orlando, et al. 2007	Gift from Ken Birnbaum/Hasslehoff collection
RM1000	Lateral root primordia initials	Transcriptome	Brady, Orlando, et al. 2007	Gift from Laurent Nussaume
J2661	Mature pericycle	Transcriptome	Levesque et al. 2006	Levesque et al.2006
APL	Phloem sieve cells and companion cells	Transcriptome	Lee et al. 2006	Lee et al.2006
CORTEX	Cortex	Transcriptome	Lee et al. 2006	Lee et al.2006
S18	Differentiating xylem	Transcriptome	Lee et al. 2006	Lee et al.2006
35S	Root proliferating cells	Translatome	Mustroph et al. 2009	Zanetti et al. 2005
SCR	Root endodermis and Quiescent Centre	Translatome	Mustroph et al. 2009	Wysocka-Diller et al. 2000
SHR	Root vasculature	Translatome	Mustroph et al. 2009	Helariutta et al. 2000
WOL	Root vasculature	Translatome	Mustroph et al. 2009	Estelle 2001
GL2	Root atrichoblast epidermis	Translatome	Mustroph et al. 2009	Masucci et al. 1996; Szymanski et al. 1998
SULTR2	Root phloem companion cells	Translatome	Mustroph et al. 2009	Takahashi et al. 2000
CO2	Root Cortex meristamatic zone	Translatome	Mustroph et al. 2009	Heidstra et al. 2004
PEP	Root Cortex elongation and maturation zone.	Translatome	Mustroph et al. 2009	Mace et al. 2006
RPL11C	Root proliferating cells	Translatome	Mustroph et al. 2009	Williams and Sussex 1995
SUC2	Sucrose transporter	Translatome	Mustroph et al. 2009	Imlau et al. 1999

Table S2: Enriched GO-terms of coupled/uncoupled gene expression patters on translatome and transcriptome for the identical promoter data.

Coupled/ uncoupled	GO-term	Term description	No. of genes	*p*-value
C	GO:0000041	transition metal ion transport	9	3.26E-02
C	GO:0000278	mitotic cell cycle	4	1.36E-02
C	GO:0006184	GTP catabolic process	4	2.73E-02
C	GO:0006355	regulation of transcription, DNA-dependent	77	3.31E-02
C	GO:0006468	protein phosphorylation	51	1.71E-02
C	GO:0006633	fatty acid biosynthetic process	8	4.04E-02
C	GO:0006661	phosphatidylinositol biosynthetic process	8	2.35E-02
C	GO:0006807	nitrogen compound metabolic process	4	1.75E-02
C	GO:0006857	oligopeptide transport	10	8.20E-03
C	GO:0006970	response to osmotic stress	10	2.16E-02
C	GO:0006995	cellular response to nitrogen starvation	3	4.40E-02
C	GO:0007169	transmembrane receptor protein tyrosine kinase signaling	10	2.51E-02
C	GO:0008610	lipid biosynthetic process	4	1.36E-02
C	GO:0009416	response to light stimulus	11	4.93E-02
C	GO:0009553	embryo sac development	5	3.15E-02
C	GO:0009611	response to wounding	20	1.92E-02
C	GO:0009617	response to bacterium	12	2.08E-02
C	GO:0009741	response to brassinosteroid stimulus	7	2.23E-02
C	GO:0009834	secondary cell wall biogenesis	5	4.00E-03
C	GO:0009862	systemic acquired resistance, salicylic acid med. sign. pathway	17	2.35E-02
C	GO:0009886	post-embryonic morphogenesis	3	3.09E-02
C	GO:0009908	flower development	8	1.62E-02
C	GO:0009939	positive regulation of gibberellic acid mediated signaling	3	3.26E-04
C	GO:0010014	meristem initiation	10	2.51E-02
C	GO:0010073	meristem maintenance	6	3.97E-02
C	GO:0010089	xylem development	9	3.99E-03
C	GO:0010150	leaf senescence	7	6.88E-03
C	GO:0010162	seed dormancy process	11	2.68E-02
C	GO:0010167	response to nitrate	15	9.78E-03
C	GO:0010260	organ senescence	4	1.75E-02
C	GO:0010440	stomatal lineage progression	5	4.87E-02
C	GO:0010583	response to cyclopentenone	12	1.30E-02
C	GO:0015706	nitrate transport	15	1.57E-02
C	GO:0016569	covalent chromatin modification	3	2.02E-02
C	GO:0042218	1-aminocyclopropane-1-carboxylate biosynthetic process	3	3.09E-02
C	GO:0042546	cell wall biogenesis	7	1.06E-02
C	GO:0043069	negative regulation of programmed cell death	13	1.28E-02
C	GO:0045454	cell redox homeostasis	12	3.69E-03
C	GO:0046855	inositol phosphate dephosphorylation	3	3.94E-02
U	GO:0006486	protein glycosylation	7	8.04E-03
U	GO:0007010	cytoskeleton organization	5	4.61E-02
U	GO:0009607	response to biotic stimulus	4	1.63E-03
U	GO:0009860	pollen tube growth	8	4.26E-02
U	GO:0009886	post-embryonic morphogenesis	3	3.41E-03
U	GO:0016579	protein deubiquitination	3	4.22E-02
U	GO:0048364	root development	7	3.89E-03
U	GO:0048366	leaf development	5	3.95E-02
U	GO:0048440	carpel development	4	3.43E-02
U	GO:0048507	meristem development	3	2.41E-02
U	GO:0048653	anther development	4	1.20E-02
U	GO:0048825	cotyledon development	5	6.47E-03
U	GO:0051301	cell division	5	1.22E-02

Table S3: Enriched GO-terms of coupled/uncoupled gene expression patters on translatome and transcriptome for the common promoters.

Coupled/ uncoupled	GO-term	Term description	No. of genes	*p*-value
C	GO:0000023	maltose metabolic process	10	2.55E-02
C	GO:0000041	transition metal ion transport	9	1.53E-02
C	GO:0000956	nuclear-transcribed mRNA catabolic process	12	1.09E-04
C	GO:0006355	regulation of transcription, DNA-dependent	68	3.73E-02
C	GO:0006399	tRNA metabolic process	4	2.61E-02
C	GO:0006468	protein phosphorylation	45	2.13E-02
C	GO:0006817	phosphate ion transport	3	3.14E-02
C	GO:0006914	Autophagy	5	4.99E-02
C	GO:0007043	cell-cell junction assembly	3	2.21E-04
C	GO:0007165	signal transduction	28	1.26E-02
C	GO:0007186	G-protein coupled receptor signaling pathway	3	4.27E-03
C	GO:0007188	adenylate cyclase-modulating G-prot. coupled receptor sign.	3	8.98E-05
C	GO:0007346	regulation of mitotic cell cycle	3	4.71E-02
C	GO:0007389	pattern specification process	5	3.21E-02
C	GO:0008219	cell death	5	3.44E-02
C	GO:0009416	response to light stimulus	11	2.17E-02
C	GO:0009630	Gravitropism	8	4.95E-02
C	GO:0009638	Phototropism	3	5.45E-03
C	GO:0009733	response to auxin stimulus	18	4.21E-02
C	GO:0009789	positive regulation of abscisic acid mediated signaling pathway	3	1.42E-02
C	GO:0009886	post-embryonic morphogenesis	3	2.18E-02
C	GO:0009902	chloroplast relocation	9	5.57E-03
C	GO:0010027	thylakoid membrane organization	13	1.91E-02
C	GO:0010051	xylem and phloem pattern formation	7	5.76E-03
C	GO:0010119	regulation of stomatal movement	4	1.96E-02
C	GO:0010413	glucuronoxylan metabolic process	16	4.81E-04
C	GO:0016114	terpenoid biosynthetic process	4	3.66E-02
C	GO:0016558	protein import into peroxisome matrix	7	3.55E-02
C	GO:0016569	covalent chromatin modification	3	1.42E-02
C	GO:0019252	starch biosynthetic process	14	4.14E-03
C	GO:0032957	inositol trisphosphate metabolic process	3	1.91E-02
C	GO:0034660	ncRNA metabolic process	9	1.26E-03
C	GO:0042545	cell wall modification	11	1.81E-03
C	GO:0045492	xylan biosynthetic process	16	4.81E-04
C	GO:0046777	protein autophosphorylation	9	3.70E-02
C	GO:0046855	inositol phosphate dephosphorylation	3	2.80E-02
C	GO:0048439	flower morphogenesis	6	2.27E-02
C	GO:0048519	negative regulation of biological process	5	1.34E-02
C	GO:0048527	lateral root development	7	1.15E-02
C	GO:0048574	long-day photoperiodism, flowering	3	1.20E-02
C	GO:2000067	regulation of root morphogenesis	3	8.98E-05
U	GO:0000902	cell morphogenesis	4	6.85E-03
U	GO:0006486	protein glycosylation	6	8.38E-03
U	GO:0006816	calcium ion transport	6	3.71E-03
U	GO:0006863	purine nucleobase transport	4	4.41E-02
U	GO:0006972	hyperosmotic response	4	3.14E-02
U	GO:0009734	auxin mediated signaling pathway	3	8.08E-03
U	GO:0010584	pollen exine formation	3	2.70E-02
U	GO:0015986	ATP synthesis coupled proton transport	3	2.82E-03
U	GO:0016049	cell growth	7	6.60E-04
U	GO:0030243	cellulose metabolic process	3	6.31E-03
U	GO:0044237	cellular metabolic process	3	3.83E-02
U	GO:0048868	pollen tube development	3	2.34E-02
U	GO:0080022	primary root development	4	3.32E-05

Table S4: GSEA of GO-BP terms for AEC genes for identical promoter data. A **+** indicates a high degree of conservation of co-expression relationships derived by *Z*-scores, while a **–** indicates divergences of co-expression relationships on the translatome and transcriptome.

AEC	GO-term	Term description	No. of genes	*p*-value
+	GO:0006355	regulation of transcription, DNA-dependent	12	4.47E-03
+	GO:0030968	endoplasmic reticulum unfolded protein response	3	1.52E-02
+	GO:0042546	cell wall biogenesis	3	8.20E-04
+	GO:0055085	transmembrane transport	4	4.53E-02
+	GO:0071555	cell wall organization	3	6.27E-03
-	GO:0000338	protein deneddylation	5	7.53E-03
-	GO:0000956	nuclear-transcribed mRNA catabolic process	57	1.07E-03
-	GO:0002679	respiratory burst involved in defense response	79	8.54E-07
-	GO:0006091	generation of precursor metabolites and energy	64	1.45E-21
-	GO:0006120	mitochondrial electron transport, NADH to ubiquinone	5	3.22E-02
-	GO:0006353	DNA-dependent transcription, termination	3	4.03E-02
-	GO:0006354	DNA-dependent transcription, elongation	91	2.25E-12
-	GO:0006511	ubiquitin-dependent protein catabolic process	146	3.62E-03
-	GO:0006569	tryptophan catabolic process	47	2.71E-03
-	GO:0006826	iron ion transport	62	2.08E-02
-	GO:0006863	purine nucleobase transport	87	9.65E-16
-	GO:0006873	cellular ion homeostasis	3	4.03E-02
-	GO:0006882	cellular zinc ion homeostasis	11	4.14E-02
-	GO:0006952	defense response	169	1.46E-02
-	GO:0006974	response to DNA damage stimulus	9	3.42E-02
-	GO:0007243	intracellular protein kinase cascade	10	3.82E-02
-	GO:0007275	multicellular organismal development	67	1.89E-02
-	GO:0007346	regulation of mitotic cell cycle	18	1.37E-02
-	GO:0008272	sulfate transport	10	1.97E-02
-	GO:0008643	carbohydrate transport	6	4.34E-02
-	GO:0009150	purine ribonucleotide metabolic process	3	4.03E-02
-	GO:0009414	response to water deprivation	147	1.06E-02
-	GO:0009607	response to biotic stimulus	22	3.73E-03
-	GO:0009611	response to wounding	158	9.79E-04
-	GO:0009612	response to mechanical stimulus	38	3.17E-05
-	GO:0009615	response to virus	16	4.97E-02
-	GO:0009620	response to fungus	59	3.73E-04
-	GO:0009646	response to absence of light	16	3.25E-02
-	GO:0009653	anatomical structure morphogenesis	9	6.16E-03
-	GO:0009684	indoleacetic acid biosynthetic process	59	5.07E-03
-	GO:0009693	ethylene biosynthetic process	70	3.19E-05
-	GO:0009695	jasmonic acid biosynthetic process	70	4.54E-03
-	GO:0009753	response to jasmonic acid stimulus	135	3.55E-04
-	GO:0009767	photosynthetic electron transport chain	11	2.28E-02
-	GO:0009769	photosynthesis, light harvesting in photosystem II	5	7.53E-03
-	GO:0009809	lignin biosynthetic process	25	7.65E-03
-	GO:0009853	Photorespiration	88	1.86E-04
-	GO:0009966	regulation of signal transduction	7	2.30E-02
-	GO:0010038	response to metal ion	7	2.30E-02
-	GO:0010043	response to zinc ion	30	2.59E-02
-	GO:0010106	cellular response to iron ion starvation	58	4.14E-02
-	GO:0010167	response to nitrate	104	1.75E-03
-	GO:0010200	response to chitin	232	1.39E-08
-	GO:0010254	nectary development	3	4.03E-02
-	GO:0010262	somatic embryogenesis	5	3.22E-02
-	GO:0010286	heat acclimation	43	3.67E-03
-	GO:0010289	homogalacturonan biosynthetic process	3	4.03E-02
-	GO:0010337	regulation of salicylic acid metabolic process	6	4.34E-02
-	GO:0010387	signalosome assembly	5	3.22E-02
-	GO:0010390	histone monoubiquitination	4	1.74E-02
-	GO:0010434	bract formation	3	4.03E-02
-	GO:0010438	cellular response to sulfur starvation	4	1.74E-02

-	GO:0010507	negative regulation of autophagy	3	4.03E-02
-	GO:0010599	production of lsiRNA involved in RNA interference	3	4.03E-02
-	GO:0015675	nickel cation transport	3	4.03E-02
-	GO:0015706	nitrate transport	111	7.94E-04
-	GO:0015866	ADP transport	3	4.03E-02
-	GO:0015979	Photosynthesis	140	3.08E-12
-	GO:0015986	ATP synthesis coupled proton transport	17	7.13E-03
-	GO:0015991	ATP hydrolysis coupled proton transport	16	3.25E-02
-	GO:0015992	proton transport	12	4.40E-02
-	GO:0016106	sesquiterpenoid biosynthetic process	3	4.03E-02
-	GO:0016441	posttranscriptional gene silencing	7	2.30E-02
-	GO:0018119	peptidyl-cysteine S-nitrosylation	11	1.12E-02
-	GO:0019684	photosynthesis, light reaction	80	5.03E-03
-	GO:0019761	glucosinolate biosynthetic process	88	1.73E-03
-	GO:0030010	establishment of cell polarity	3	4.03E-02
-	GO:0030029	actin filament-based process	5	7.53E-03
-	GO:0030048	actin filament-based movement	43	2.95E-02
-	GO:0030308	negative regulation of cell growth	4	1.74E-02
-	GO:0031930	mitochondria-nucleus signaling pathway	4	1.74E-02
-	GO:0034605	cellular response to heat	9	3.42E-02
-	GO:0034755	iron ion transmembrane transport	4	1.74E-02
-	GO:0035556	intracellular signal transduction	102	1.44E-03
-	GO:0042218	1-aminocyclopropane-1-carboxylate biosynthetic	15	5.33E-03
-	GO:0042325	regulation of phosphorylation	3	4.03E-02
-	GO:0042742	defense response to bacterium	159	2.63E-02
-	GO:0042773	ATP synthesis coupled electron transport	8	6.06E-04
-	GO:0043069	negative regulation of programmed cell death	84	2.31E-02
-	GO:0043255	regulation of carbohydrate biosynthetic process	3	4.03E-02
-	GO:0045333	cellular respiration	20	2.99E-04
-	GO:0046168	glycerol-3-phosphate catabolic process	3	4.03E-02
-	GO:0046786	viral replication complex formation and maintenance	4	1.74E-02
-	GO:0048507	meristem development	24	3.62E-02
-	GO:0048510	reg. of timing of trans. from veg. to reproductive phase	14	1.72E-02
-	GO:0048523	negative regulation of cellular process	6	1.57E-02
-	GO:0048830	adventitious root development	4	1.74E-02
-	GO:0048869	cellular developmental process	7	1.40E-03
-	GO:0051026	chiasma assembly	8	2.92E-02
-	GO:0051090	regulation of sequence-specific DNA binding TF	3	4.03E-02
-	GO:0051127	positive regulation of actin nucleation	3	4.03E-02
-	GO:0051603	proteolysis involved in cellular protein catabolic	17	1.75E-03
-	GO:0051788	response to misfolded protein	95	7.25E-03
-	GO:0051865	protein autoubiquitination	9	1.71E-03
-	GO:0052542	defense response by callose deposition	26	4.90E-02
-	GO:0055062	phosphate ion homeostasis	7	2.30E-02
-	GO:0070301	cellular response to hydrogen peroxide	3	4.03E-02
-	GO:0070734	histone H3-K27 methylation	3	4.03E-02
-	GO:0071456	cellular response to hypoxia	14	4.76E-02
-	GO:0071722	detoxification of arsenic-containing substance	3	4.03E-02
-	GO:0072334	UDP-galactose transmembrane transport	3	4.03E-02
-	GO:0080003	thalianol metabolic process	3	4.03E-02
-	GO:0080027	response to herbivore	7	1.40E-03
-	GO:0080086	stamen filament development	7	7.59E-03
-	GO:0080129	proteasome core complex assembly	72	8.44E-04

Table S5: GSEA of GO-BP terms for AEC genes for the common promoters. A **+** indicates a high degree of conservation of co-expression relationships derived by *Z*-scores, while a **–** indicates divergences of co-expression relationships on the translatome and transcriptome.

**AEC**	**GO-term**	**Term description**	**No. of genes**	***p*-value**
+	GO:0000271	polysaccharide biosynthetic process	3	3.26E-04
+	GO:0007165	signal transduction	4	8.68E-03
+	GO:0007389	pattern specification process	3	8.18E-05
+	GO:0008361	regulation of cell size	3	4.52E-05
+	GO:0009825	multidimensional cell growth	3	2.47E-04
+	GO:0009926	auxin polar transport	3	1.60E-04
+	GO:0009932	cell tip growth	3	2.20E-04
+	GO:0010015	root morphogenesis	3	3.68E-05
+	GO:0010075	regulation of meristem growth	4	9.70E-05
+	GO:0010817	regulation of hormone levels	3	1.40E-04
+	GO:0040007	Growth	3	7.75E-05
+	GO:0043481	anthocyanin accumulation in tissues in response to UV light	3	6.75E-04
+	GO:0048767	root hair elongation	3	2.45E-03
+	GO:0071555	cell wall organization	3	1.04E-03
-	GO:0000041	transition metal ion transport	78	2.12E-03
-	GO:0000059	protein import into nucleus, docking	11	1.51E-02
-	GO:0000302	response to reactive oxygen species	21	2.78E-02
-	GO:0000338	protein deneddylation	5	2.45E-02
-	GO:0000461	endonucleolytic cleavage	4	4.48E-02
-	GO:0002679	respiratory burst involved in defense response	90	7.35E-06
-	GO:0006071	glycerol metabolic process	12	2.52E-02
-	GO:0006091	generation of precursor metabolites and energy	65	1.30E-16
-	GO:0006352	DNA-dependent transcription, initiation	25	2.33E-02
-	GO:0006354	DNA-dependent transcription, elongation	94	3.06E-07
-	GO:0006355	regulation of transcription, DNA-dependent	893	5.33E-03
-	GO:0006511	ubiquitin-dependent protein catabolic process	179	3.31E-03
-	GO:0006569	tryptophan catabolic process	54	1.03E-02
-	GO:0006612	protein targeting to membrane	217	3.24E-02
-	GO:0006629	lipid metabolic process	122	2.97E-02
-	GO:0006636	unsaturated fatty acid biosynthetic process	46	1.55E-02
-	GO:0006680	glucosylceramide catabolic process	4	4.48E-02
-	GO:0006814	sodium ion transport	26	1.70E-02
-	GO:0006826	iron ion transport	83	2.61E-04
-	GO:0006857	oligopeptide transport	76	2.80E-04
-	GO:0006863	purine nucleobase transport	96	8.25E-15
-	GO:0006886	intracellular protein transport	109	3.21E-02
-	GO:0006952	defense response	219	4.33E-04
-	GO:0006984	ER-nucleus signaling pathway	11	3.83E-02
-	GO:0007243	intracellular protein kinase cascade	13	5.80E-03
-	GO:0008272	sulfate transport	11	3.83E-02
-	GO:0009228	thiamine biosynthetic process	7	3.46E-02
-	GO:0009395	phospholipid catabolic process	5	2.45E-02
-	GO:0009414	response to water deprivation	177	3.24E-02
-	GO:0009607	response to biotic stimulus	24	1.85E-02
-	GO:0009611	response to wounding	192	1.32E-03
-	GO:0009612	response to mechanical stimulus	44	2.13E-05
-	GO:0009620	response to fungus	69	7.64E-04
-	GO:0009637	response to blue light	64	4.24E-03
-	GO:0009646	response to absence of light	23	1.50E-04
-	GO:0009649	entrainment of circadian clock	5	2.45E-02
-	GO:0009653	anatomical structure morphogenesis	9	3.80E-02
-	GO:0009684	indoleacetic acid biosynthetic process	68	2.48E-02
-	GO:0009693	ethylene biosynthetic process	80	2.37E-04
-	GO:0009694	jasmonic acid metabolic process	25	2.33E-02
-	GO:0009695	jasmonic acid biosynthetic process	92	3.40E-05
-	GO:0009734	auxin mediated signaling pathway	26	1.70E-02
-	GO:0009751	response to salicylic acid stimulus	81	1.93E-02
-	GO:0009753	response to jasmonic acid stimulus	164	2.92E-04

-	GO:0009767	photosynthetic electron transport chain	13	1.64E-02
-	GO:0009768	photosynthesis, light harvesting in photosystem I	4	4.48E-02
-	GO:0009769	photosynthesis, light harvesting in photosystem II	5	2.45E-02
-	GO:0009773	photosynthetic electron transport in photosystem I	36	1.73E-02
-	GO:0009813	flavonoid biosynthetic process	40	1.41E-02
-	GO:0009867	jasmonic acid mediated signaling pathway	170	4.44E-03
-	GO:0009939	positive regulation of gibberellic acid mediated signaling	5	2.45E-02
-	GO:0010043	response to zinc ion	37	1.31E-02
-	GO:0010093	specification of floral organ identity	24	1.01E-02
-	GO:0010103	stomatal complex morphogenesis	89	1.30E-02
-	GO:0010106	cellular response to iron ion starvation	78	9.66E-04
-	GO:0010107	potassium ion import	4	4.48E-02
-	GO:0010114	response to red light	59	2.56E-02
-	GO:0010167	response to nitrate	130	2.35E-04
-	GO:0010200	response to chitin	286	9.33E-11
-	GO:0010207	photosystem II assembly	111	2.51E-03
-	GO:0010231	maintenance of seed dormancy	4	4.48E-02
-	GO:0010286	heat acclimation	52	2.03E-03
-	GO:0010363	regulation of plant-type hypersensitive response	220	2.36E-02
-	GO:0010390	histone monoubiquitination	4	4.48E-02
-	GO:0010438	cellular response to sulfur starvation	4	4.48E-02
-	GO:0015706	nitrate transport	137	1.97E-04
-	GO:0015979	Photosynthesis	164	6.86E-13
-	GO:0018119	peptidyl-cysteine S-nitrosylation	12	2.52E-02
-	GO:0019684	photosynthesis, light reaction	105	4.07E-05
-	GO:0019761	glucosinolate biosynthetic process	101	1.96E-02
-	GO:0030308	negative regulation of cell growth	4	4.48E-02
-	GO:0031540	regulation of anthocyanin biosynthetic process	12	2.52E-02
-	GO:0031930	mitochondria-nucleus signaling pathway	4	4.48E-02
-	GO:0034755	iron ion transmembrane transport	4	4.48E-02
-	GO:0035556	intracellular signal transduction	125	6.82E-04
-	GO:0035725	sodium ion transmembrane transport	31	1.75E-02
-	GO:0042218	1-aminocyclopropane-1-carboxylate biosynthetic process	17	7.30E-03
-	GO:0042549	photosystem II stabilization	7	7.33E-03
-	GO:0042773	ATP synthesis coupled electron transport	7	3.46E-02
-	GO:0043161	proteasomal ubiquitin-dependent protein catabolic process	73	3.69E-02
-	GO:0043407	negative regulation of MAP kinase activity	12	9.39E-03
-	GO:0045333	cellular respiration	20	1.10E-02
-	GO:0045727	positive regulation of translation	13	1.46E-03
-	GO:0046786	viral replication complex formation and maintenance	4	4.48E-02
-	GO:0048235	pollen sperm cell differentiation	20	2.15E-02
-	GO:0048869	cellular developmental process	7	7.33E-03
-	GO:0050832	defense response to fungus	177	2.77E-02
-	GO:0050994	regulation of lipid catabolic process	4	4.48E-02
-	GO:0051603	proteolysis involved in cellular protein catabolic process	18	1.12E-02
-	GO:0051865	protein autoubiquitination	9	1.23E-02
-	GO:0052542	defense response by callose deposition	32	3.26E-02
-	GO:0055062	phosphate ion homeostasis	8	2.07E-02
-	GO:0055114	oxidation-reduction process	635	4.09E-02
-	GO:0070838	divalent metal ion transport	53	4.67E-02
-	GO:0071216	cellular response to biotic stimulus	11	3.83E-02
-	GO:0071577	zinc ion transmembrane transport	10	2.41E-02
-	GO:0071786	endoplasmic reticulum tubular network organization	5	2.45E-02
-	GO:0080027	response to herbivore	7	7.33E-03
-	GO:0080086	stamen filament development	7	3.46E-02
-	GO:0080129	proteasome core complex assembly	80	2.31E-02
-	GO:0090333	regulation of stomatal closure	7	3.46E-02
-	GO:2000037	regulation of stomatal complex patterning	5	2.45E-02
-	GO:2000038	regulation of stomatal complex development	5	2.45E-02
-	GO:2001141	regulation of RNA biosynthetic process	5	2.45E-02

Table S6: All possible motif occurences across the identical promoters of transcriptome and translatome. The observed motifs are depicted in the order mentioned in the third column header. A characteristic pattern of the pairwise differences are represented by 1 (significant mean difference of expression values) and 0(no significant mean difference of expression values).

**System level**	**Number of genes**	**Observed motif** (GL2-SCR, GL2-WOL,SUC2-GL2,SUC2-SCR, SUC2-WOL, WOL-SCR)	***p*-value**
Transcriptome	214	001110	0.00
Transcriptome	58	011111	0.00
Transcriptome	47	001000	0.00
Transcriptome	33	011101	0.00
Transcriptome	34	111000	0.00
Transcriptome	31	111110	0.00
Transcriptome	22	110110	0.00
Transcriptome	19	100100	0.04
Transcriptome	26	100000	0.20
Transcriptome	25	100101	0.02
Transcriptome	23	010011	0.07
Transcriptome	25	001010	0.99
Transcriptome	67	000000	1.00
Transcriptome	25	001100	1.00
Transcriptome	16	011110	1.00
Translatome	203	100101	0.00
Translatome	94	001110	0.00
Translatome	72	100100	0.00
Translatome	46	010011	0.00
Translatome	28	101011	0.00
Translatome	29	110110	0.01
Translatome	45	101110	0.11
Translatome	37	000110	0.11
Translatome	8	111011	0.92
Translatome	57	100110	0.95
Translatome	23	101111	0.99
Translatome	12	000100	0.99
Translatome	10	111000	0.99
Translatome	21	111111	1.00

Table S7: Enriched GO-terms of gene with charecteristic cell type specific gene expression patters found for motif 1 and motif 2.

Motif	GO-term	Term description	No. of genes	*p*-value
1	GO:0001666	response to hypoxia	4	1.03E-02
1	GO:0005986	sucrose biosynthetic process	3	1.31E-03
1	GO:0006084	acetyl-CoA metabolic process	5	2.44E-03
1	GO:0006085	acetyl-CoA biosynthetic process	3	2.02E-04
1	GO:0006612	protein targeting to membrane	9	4.85E-02
1	GO:0006810	Transport	9	4.45E-02
1	GO:0006833	water transport	5	3.38E-02
1	GO:0008033	tRNA processing	3	4.79E-03
1	GO:0008219	cell death	3	2.59E-02
1	GO:0009684	indoleacetic acid biosynthetic process	4	3.74E-02
1	GO:0009691	cytokinin biosynthetic process	3	5.25E-03
1	GO:0009735	response to cytokinin stimulus	3	2.72E-02
1	GO:0009736	cytokinin mediated signaling pathway	3	4.74E-02
1	GO:0009741	response to brassinosteroid stimulus	4	1.19E-02
1	GO:0009744	response to sucrose stimulus	9	1.29E-03
1	GO:0009749	response to glucose stimulus	6	3.83E-04
1	GO:0009750	response to fructose stimulus	6	9.06E-03
1	GO:0009910	negative regulation of flower development	3	1.37E-02
1	GO:0009963	positive regulation of flavonoid biosynthetic process	6	1.79E-03
1	GO:0010075	regulation of meristem growth	5	3.93E-02
1	GO:0010264	myo-inositol hexakisphosphate biosynthetic process	3	3.37E-02
1	GO:0016036	cellular response to phosphate starvation	5	1.87E-02
1	GO:0016126	sterol biosynthetic process	7	4.71E-03
1	GO:0016132	brassinosteroid biosynthetic process	4	4.57E-02
1	GO:0019375	galactolipid biosynthetic process	5	7.54E-03
1	GO:0019745	pentacyclic triterpenoid biosynthetic process	3	1.06E-02
1	GO:0045454	cell redox homeostasis	9	3.59E-05
1	GO:0048653	anther development	7	6.87E-06
1	GO:0051645	Golgi localization	3	1.21E-02
1	GO:0051646	mitochondrion localization	3	1.21E-02
1	GO:0055085	transmembrane transport	15	8.51E-04
1	GO:0060151	peroxisome localization	3	1.21E-02
2	GO:0000041	transition metal ion transport	11	5.21E-08
2	GO:0006084	acetyl-CoA metabolic process	6	1.51E-04
2	GO:0006633	fatty acid biosynthetic process	5	3.98E-03
2	GO:0006826	iron ion transport	6	1.50E-03
2	GO:0006865	amino acid transport	4	4.78E-02
2	GO:0006979	response to oxidative stress	12	1.50E-05
2	GO:0007043	cell-cell junction assembly	5	1.00E-10
2	GO:0007169	transmembrane receptor protein tyrosine kinase sign.	7	4.11E-04
2	GO:0007389	pattern specification process	3	1.57E-02
2	GO:0008152	metabolic process	16	2.67E-02
2	GO:0008361	regulation of cell size	3	9.11E-03
2	GO:0009269	response to desiccation	3	4.55E-03
2	GO:0009611	response to wounding	10	1.92E-03
2	GO:0009664	plant-type cell wall organization	4	4.05E-02
2	GO:0009739	response to gibberellin stimulus	5	3.06E-03
2	GO:0009741	response to brassinosteroid stimulus	5	1.01E-03
2	GO:0009750	response to fructose stimulus	5	1.62E-02
2	GO:0009805	coumarin biosynthetic process	4	1.84E-03
2	GO:0009825	multidimensional cell growth	3	4.15E-02
2	GO:0009926	auxin polar transport	3	2.85E-02
2	GO:0009932	cell tip growth	4	7.52E-03
2	GO:0009963	positive regulation of flavonoid biosynthetic process	6	7.36E-04
2	GO:0010015	root morphogenesis	3	7.54E-03
2	GO:0010075	regulation of meristem growth	7	1.20E-03
2	GO:0010106	cellular response to iron ion starvation	6	1.20E-03
2	GO:0010167	response to nitrate	10	5.37E-05
2	GO:0010413	glucuronoxylan metabolic process	8	7.10E-04
2	GO:0010817	regulation of hormone levels	3	2.54E-02

2	GO:0015706	nitrate transport	10	8.54E-05
2	GO:0016126	sterol biosynthetic process	9	7.11E-05
2	GO:0016132	brassinosteroid biosynthetic process	10	4.23E-07
2	GO:0030003	cellular cation homeostasis	4	1.96E-02
2	GO:0040007	Growth	3	1.49E-02
2	GO:0042545	cell wall modification	8	3.30E-05
2	GO:0045492	xylan biosynthetic process	8	7.10E-04
2	GO:0048765	root hair cell differentiation	5	1.62E-02
2	GO:0048767	root hair elongation	6	1.13E-02
2	GO:0055114	oxidation-reduction process	22	7.23E-03
2	GO:0070838	divalent metal ion transport	4	9.36E-03
